# Methanesulfonic acid (MSA) in clean processes and applications: a tutorial review

**DOI:** 10.1039/d4gc02031f

**Published:** 2024-06-27

**Authors:** Koen Binnemans, Peter Tom Jones

**Affiliations:** a KU Leuven, Department of Chemistry Celestijnenlaan 200F P.O. box 2404 B-3001 Heverlee Belgium Koen.Binnemans@kuleuven.be; b KU Leuven, Department of Materials Engineering Kasteelpark Arenberg 44 bus 2450 B-3001 Heverlee Belgium

## Abstract

This Tutorial Review acquaints chemists and metallurgists with the properties and industrial applications of methanesulfonic acid (MSA, CH_3_SO_3_H). Over the past quarter-century, MSA has garnered increasing interest as a reagent for green chemistry due to its strong acidity, while circumventing many of the challenges associated with handling concentrated sulfuric acid, hydrochloric acid, or nitric acid. Concentrated MSA is a non-oxidizing reagent, exhibiting high chemical stability against redox reactions and hydrolysis, as well as high thermal stability and limited corrosivity towards construction materials. It is colorless, odorless, and possesses a very low vapor pressure. MSA combines commendable biodegradability with low toxicity. It is extensively utilized as a Brønsted acid catalyst for esterification or alkylation reactions, and is employed in biodiesel production. The high solubility of its metal salts, the high electrical conductivity of its concentrated solutions, coupled with the high electrochemical stability of MSA and its anion, make MSA-based electrolytes beneficial in electrochemical applications. Examples include the electrodeposition of tin–lead solder for electronic applications and the high-speed plating of tin on steel plate for food cans. MSA-based electrolytes are used in redox flow batteries (RFBs). MSA offers a much safer and environmentally friendlier alternative to electrolytes based on fluoroboric or fluorosilicic acid. A novel application area is as a strong acid in extractive metallurgy, where it may contribute to the development of circular hydrometallurgy. MSA is being explored in lithium-ion battery recycling flowsheets, as well as in other applications in the field of metal recovery and refining. However, this review is not solely about the advantages of MSA for green chemistry or clean technologies, as there are also some potential drawbacks. Apart from its higher price compared to regular strong acids, MSA has only minor advantages for applications where sulfuric acid performs well. Since methanesulfonate biodegrades into sulfate, the same emission restrictions as for sulfate should be considered. In conclusion, MSA is the acid of choice for applications where metal sulfates cannot be used due to poor solubility or where concentrated sulfuric acid is too reactive towards organics.

## Abstract 2

Methanesulfonic acid (MSA) bridges organic and inorganic green chemistry since it is a strong non-oxidizing organic acid with a very low vapor pressure and forming highly soluble metal salts.

## Graphical abstract

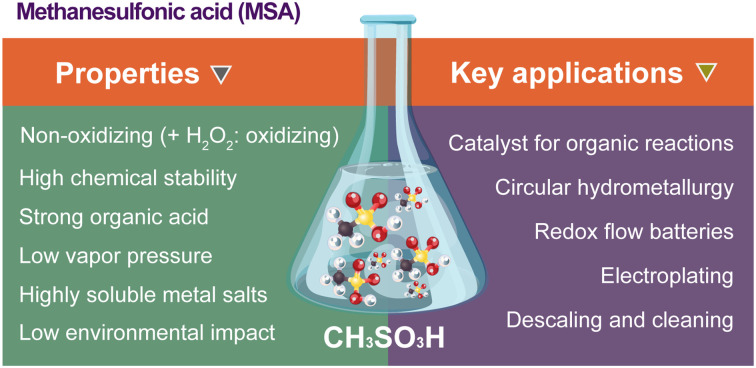

## Introduction

Methanesulfonic Acid (MSA), possessing the chemical formula CH_3_SO_3_H, is the smallest constituent of the alkanesulfonic acids family. MSA is alternatively referred to as methylsulfonic acid or mesylic acid. Throughout this review, the abbreviation MSA will be employed. Salts of MSA are recognized as methanesulfonates or mesylates, occasionally abbreviated to MsO or OMs. MSA serves as a bridge between organic and inorganic chemistry. With a single carbon atom in its methyl group, it can be categorized as a C1 compound in organic chemistry. MSA is not only completely miscible with water but also with oxygenated organic solvents such as alcohols and diethyl ether. Regarding its chemical reactivity, MSA bears more resemblance to inorganic compounds than to organic ones. The methyl group exhibits low chemical reactivity, and MSA is a significantly stronger acid than the organic carboxylic acids, boasting acid strengths comparable to those of the strong inorganic acids. Considering that MSA can be formally regarded as a substituted sulfuric acid, formed by substituting an OH group in H_2_SO_4_ with a CH_3_ group, it is unsurprising that MSA and sulfuric acid share similar chemical properties. Conversely, while concentrated sulfuric acid is strongly oxidizing, concentrated MSA is not an oxidizing acid. From a historical perspective, MSA serves as a link between organic and inorganic chemistry. In the mid-19th century, the German chemist Hermann Kolbe utilized MSA as one of the examples to illustrate that there are no fundamental differences between organic and inorganic compounds, and the synthesis of MSA contributed to the abandonment of the vitalism theory in organic chemistry. In 1845, Kolbe obtained MSA *via* the reductive dehalogenation of trichloromethanesulfonic acid.^1^ This is one of the oldest examples of electroorganic synthesis.

From 1845 to the conclusion of the 20th century, MSA attracted only peripheral interest from researchers, primarily due to the reluctance of commercial suppliers to ensure widespread availability. Noteworthy publications include the study by Billeter (1905),^2^ which reports the boiling point of 167 °C at a pressure of 10 mm Hg, and that of Berthoud (1929),^3^ which serves as the original source of several of the thermodynamic properties of MSA, such as its mass density, the melting point of +20 °C, and the MSA–water binary phase diagram.

MSA became more accessible following the development of an industrial synthesis process by the Standard Oil Company of Indiana at the conclusion of the 1940s.^4^ Nonetheless, it was not until the early 1980s that a substantial market for MSA was established, with applications including electrolytes for the electroplating of tin–lead solder in the electronics industry, other electrochemical processes involving tin and lead, its use as an esterification or alkylation catalyst in the chemical industry, and as a solvent for polymers that are difficult to dissolve. These represented niche applications that consumed relatively small volumes of MSA. For use in the microelectronics industry, MSA of an exceptionally high purity was made available. MSA remained largely unrecognized by academic chemists until the publication of a paper by Gernon and colleagues from the Elf Atochem company in 1999.^5^ MSA was portrayed as a green acid primarily because it offers a much safer and environmentally friendlier alternative to fluoroboric and fluorosilicic acid in electrochemical applications such as the electrodeposition of tin–lead solder on electronic devices, the electroplating of tin on steel plate for food cans, and the electrowinning of lead. MSA received scant attention in organic chemistry, although it may also present advantages from a sustainability perspective.

This Tutorial Review on MSA and its industrial applications offers a comprehensive overview of the advancements in MSA chemistry and its applications, with a focus on the cleantech sector. The review will demonstrate how MSA could facilitate the ongoing evolution of green chemistry and sustainable technologies. The intended audience comprises chemists and metallurgists who are keen to expand their knowledge on MSA, its properties, and applications. Our address is not limited to early-career researchers, but also extends to more seasoned individuals, as we acknowledge that MSA remains a largely unfamiliar chemical within the scientific community. This review serves as a complement to another recent review penned by the authors, which delineates the benefits and applications of MSA in hydrometallurgy.^6^ We will discuss not only the advantages of MSA, but we will also highlight its limitations, enabling the reader to make an objective evaluation of MSA's potential as a green reagent.

## Industrial synthesis processes

A prerequisite for the extensive utilization of MSA is its large-scale production and ready availability at a cost-effective price. Furthermore, it cannot be designated as a green acid if its synthesis does not adhere to environmentally friendly processes. For a considerable duration, these conditions were not met. MSA was classified more as a specialty chemical rather than a commodity one, and it was produced for a small, niche market, rendering it relatively expensive. Its industrial production processes did not exemplify best practices in green chemistry. Over the past 25 years, the MSA industry has witnessed significant improvements in the synthesis process. The direct reaction between methane and sulfur trioxide represents a new process conducted under relatively mild conditions and with 100% atomic economy. The current global production capacity amounts to several tens of thousands of metric tons. Although MSA will never be as inexpensive as sulfuric acid, its price is already on par with that of other bulk organic chemicals. In this section, we provide an overview of the industrial processes that have been developed, or are currently under development, for the large-scale production of MSA.

### Air oxidation process

The first industrial process for MSA production was developed in the 1940s by the Standard Oil Company of Indiana (USA).^4^ This process is now obsolete and was based on the NO_*x*_-based air oxidation of methanethiol (methyl mercaptan, CH_3_SH) or dimethyl disulfide (DMDS; CH_3_S–SCH_3_), followed by a stripping procedure to remove the residual NO_*x*_ from the MSA product. The chemical reactions are:

1 2CH_3_SH + 3O_2_ → 2CH_3_SO_3_H

2 2CH_3_S–SCH_3_ + 5O_2_ + 2H_2_O → 4CH_3_SO_3_H

Although it was a cheap process (using air as a reagent), it suffered from a poor product purity and explosion hazards.

### Chlorine oxidation process

In 1967, Pennwalt Corporation (USA) disclosed a two-step process based on the direct chlorine oxidation of an emulsion of methanethiol in a concentrated aqueous HCl solution to form methanesulfonyl chloride, which could be hydrolyzed to MSA. The two main reactions are:

3 CH_3_SH + 3Cl_2_ + 2H_2_O → CH_3_SO_2_Cl + 5HCl

4 CH_3_SO_2_Cl + H_2_O → CH_3_SO_3_H + HCl

The overall reaction is:

5 CH_3_SH + 3Cl_2_ + 3H_2_O → CH_3_SO_2_H + 6HCl

The chlorination reaction to form methanesulfonyl chloride gives very high conversion yields, and chlorine gas must be present in amounts ranging from a stoichiometric level to an excess of 5% based on the amount of methyl mercaptan feed.^7^ The reaction can be carried out at ambient temperatures and pressures. The aqueous hydrochloric acid solution is instrumental in moderating the exothermic reaction. Although this process is highly efficient, it produces 6 moles of HCl per mole of MSA, resulting in a coupling of the demand for the primary product MSA and the byproduct hydrochloric acid. The process necessitates the manipulation of hazardous chlorine gas and corrosive hydrochloric acid. The participation of chlorine results in elevated chloride concentrations in the end product. Overoxidation has the potential to generate sulfuric acid and sulfate impurities. These chloride and sulfate impurities may pose a problem for MSA applications that demand high purity, such as those in the microelectronics sector. Incomplete oxidation of methanethiol can result in the production of malodorous organosulfur impurities in the final MSA product. These malodorous impurities can be eliminated through treatment with ozone gas.^8^ The process of chlorine oxidation continues to be employed by Arkema SA, a French company, for the production of MSA.

### BASF process

The chemical company BASF SE (Germany) developed a chlorine-free process for MSA production.^9^ In a first step, an excess of H_2_S gas reacts with methanol (in a 3 : 2 molar ratio) to form methanethiol (CH_3_SH) in 98% yield, by using a potassium tungstate and aluminum oxide catalyst:

6 CH_3_OH + H_2_S → CH_3_SH + H_2_O

Methanol is obtained by conversion of syngas (a H_2_/CO mixture, nowadays obtained from natural gas). Methanethiol reacts further with elemental sulfur, in the presence of a secondary amine as the main catalyst. The reaction products are dimethyl disulfide (DMDS) and H_2_S, which can be reused in the first reaction:

7 2CH_3_SH + S → CH_3_S–SCH_3_ + H_2_S

Excess H_2_S is burned in a Claus process used for gas desulfurization, to produce elemental sulfur, which can in turn be reused for the DMDS reaction. The yield is about 90%, with polysulfides (CH_3_S_*x*_CH_3_) being the main side products. The DMDS is refined by distillation. The DMDS is brought into a reactor where it reacts with excess amounts of nitric acid to form MSA (in a 1 : 5 molar ratio):

8 CH_3_S–SCH_3_ + *x*HNO_3_ → 2CH_3_SO_3_H + *y*NO_*x*_

Nitrogen oxide (NO_*x*_) byproducts are directed into a nitric acid generation column to reform HNO_3_ for further reactions. Concurrently, the remaining products and byproducts are channeled into a series of two vacuum distillation columns to separate MSA from the other substances. The first column operates at temperatures ranging from 180–190 °C and pressures between 95–100 mbar. Within this column, nitric acid, water, and nitrogen oxides (which are generated in the column) are separated from the remaining products and byproducts. Minor quantities of sulfuric acid and MSA methyl ester are produced within the column, and this mixture, along with the MSA product and trace amounts of water, is directed to a second vacuum column. This subsequent column operates at temperatures of 180–190 °C and pressures of 5–10 mbar. It separates the pure MSA product, a byproduct mixture of MSA and sulfuric acid (in a 20 : 80 mass ratio), and the MSA methyl ester. The sulfuric acid is combined with a portion of the MSA and is considered a byproduct. The MSA methyl ester is treated as a waste product. The final product is MSA with a purity of 99.5%, containing very low levels of impurities, typically <1 ppm chlorine, <20 ppm sulfate, and <1 ppm metal ions. A majority of this highly pure MSA is diluted with water to yield a 70 wt% aqueous solution, which is the most commonly utilized MSA product. In 2003, BASF initiated the production of highly pure MSA in a continuous process at its facility in Ludwigshafen, Germany. BASF markets MSA as a 70% aqueous solution under the brand name Lutropur® MSA. Anhydrous MSA (99.5%) is available under the brand name Lutropur® MSA 100.

### Direct sulfonation of methane

The direct sulfonation of methane (CH_4_) is the most ideal synthesis route to MSA:

9 CH_4_ + SO_3_ → CH_3_SO_3_H

This reaction is attractive because of its 100% atom economy; all the atoms of the reagents end up in the reaction product, so that no waste byproducts are formed.^10^ The reagents consist of cost-effective and abundant resources. Methane, the primary constituent of natural gas, is available in vast amounts. However, methane derived from biogas is favored due to its renewable nature. Sulfur trioxide (SO_3_) can be synthesized *via* the catalytic oxidation of sulfur dioxide (SO_2_). Sulfur dioxide can be generated through the combustion of elemental sulfur, but it is also a byproduct of the metallurgical industry, produced during the pyrometallurgical processing of sulfide ores and concentrates.

Direct methane sulfonation appears to be a straightforward reaction, yet its application in an economically viable industrial process presents a significant challenge due to the complexities associated with methane activation.^11^ Methane exhibits low intrinsic reactivity, making it one of the most challenging molecules to functionalize in a controlled manner.^12^ The reactions typically employed for methane functionalization generate copious amounts of undesired byproducts. It was only approximately 25 years ago that it was demonstrated that methane could be sulfonated with SO_3_ in strong acid solvents using a free-radical initiator. These reactions were commonly conducted at elevated temperatures in concentrated sulfuric acid or *oleum* (a form of sulfuric acid that already contains an excess of SO_3_, also referred to as *fuming sulfuric acid*). Examples of free-radical initiators are potassium persulfate (K_2_S_2_O_8_), potassium peroxydiphosphate (K_4_P_2_O_8_), potassium superoxide (KO_2_), calcium peroxide (CaO_2_), and mixtures of urea ((NH_2_)_2_CO) and hydrogen peroxide (H_2_O_2_).^13–15^

The issues associated with these initiators include the fact that they are consumed and the inability to recycle them. Furthermore, catalysts required for these reactions include salts of precious metals, such as palladium and rhodium, as well as toxic mercury salts. These reactions are characterized by low yields due to free-radical recombination (for instance, the formation of ethane through the coupling of two methyl radicals) and unwanted side reactions, rendering them unsuitable for large-scale industrial production.

In 2016, the German chemical company GRILLO-Werke AG announced that it had developed an innovative process enabling the synthesis of MSA by direct activation of methane: the *Grillo-Methane-Sulfonation* (GMS) process.^16,17^ Oleum was treated with methane at a pressure of about 100 bar and a temperature of 50 °C, in the presence of less than 1 mol% of an electrophilic initiator. The initiator contains various sulfonyl peroxide derivatives prepared as a mixture of MSA, H_2_O_2_ and oleum.^18^ Monomethylsulfonyl peroxide sulfuric acid, HOS(O)_2_OOS(O)_2_CH_3_ (MMSP), is the reactive species. Advantages of the GMS process are that it can be operated at a moderate temperature, no metal catalysts are required and only MSA is formed. There are no side products. The disadvantage is that a relatively high methane pressure is required to accelerate the reaction.

**Fig. 1:**
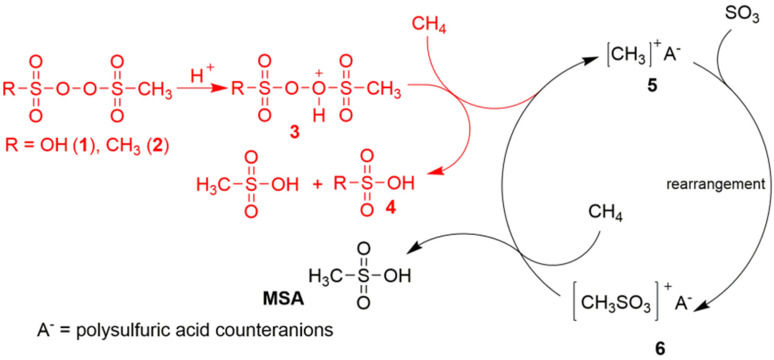
Proposed ionic reaction mechanism for the C–H activation of methane in the selective production of MSA in the Grillo-Methane-Sulfonation (GMS) process. Reproduced from ref. 19 with permission from the American Association for the Advancement of Science (AAAS), copyright 2019.

Díaz-Urrutia and Ott suggested that MSA is produced in the GMS process through a cationic chain reaction mechanism, rather than through a free-radical mechanism: the electrophilic sulfonyl peroxide initiator is protonated under superacidic conditions to a peroxonium ion, which creates a transient electrophilic species of unknown structure by hydride abstraction from methane.^19^ Reaction with the S=O bond of SO_3_ at the sulfur atom then produces the CH_3_S(O)_2_O^+^ intermediate, which in turn is so electrophilic that it can abstract a hydride from methane to form MSA, regenerating in this way the methyl cation for the next catalytic cycle (Fig. 1). Contrary to prior studies, the GMS process employs superacidic environments at reduced temperatures and devoid of additives, thereby establishing reaction conditions that promote the protonation of the –O–O– bond and the ensuing generation of electrophilic species that contribute to the formation of MSA. The likelihood of a radical mechanism is diminished due to the lack of a radical initiator and the observation that no higher alkanes (through radical recombination) are detected.^20^ Certain researchers have voiced skepticism regarding this mechanism due to the anticipated high reactivity of the methyl cation with the solvent, which would preclude it from serving as an intermediate in the reaction. Additional concerns include the inadequate nucleophilicity of the sulfur atom in SO_3_, and the high energy of the CH_3_–S(O)_2_O^+^ species above the starting mixture of CH_3_^+^/SO_3_.^21^ Also a protonated radical mechanism, involving the MSA cationic radical CH_3_–S(O)_2_OH˙^+^, has been proposed.^20^

**Fig. 2:**
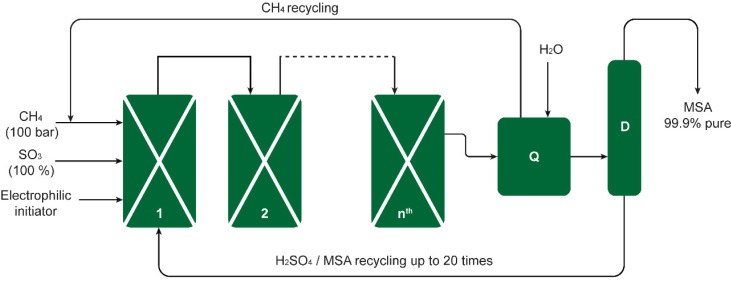
Schematic of the Grillo-Methane-Sulfonation (GMS) process. The reaction proceeds as a cascade through reactors connected in series. The pilot plant could produce up to 20 metric tons of MSA per year. The excess SO_3_ is quenched in reactor Q, the CH_4_ excess stream and the MSA/H_2_SO_4_ sump stream are recycled back to reactor 1, and the MSA-enriched mixture is distilled in column D to obtain pure MSA. Redrawn from ref. 19 with permission from the American Association for the Advancement of Science (AAAS), copyright 2019.

The Grillo process was successfully upscaled from laboratory bench scale to industrial pilot scale. The process was demonstrated in a pilot plant consisting of four reactors, producing 200 kg of pure MSA per week. This amounted to 2.3 metric tons during a campaign of 80 days (Fig. 2).^19^ Long-term reproducibility and selectivity were noted in the pilot plant, interpreted by Díaz-Urrutia and Ott as evidence for an electrophilic mechanism rather than a free-radical one for an electrophilic mechanism as opposed to a free-radical one. It was determined that the conditions of the reaction exerted a significant impact on the product distribution. For instance, at temperatures in excess of 50 °C and SO_3_ concentrations of more than 36 wt%, MSA was dehydrated to its anhydride CH_3_S(O_2_)OS(O_2_)CH_3_, which was subsequently converted in the hot oleum solution to methyl hydrogen sulfate (methyl bisulfate).^22^

In 2019, BASF procured the intellectual property rights for the direct methane sulfonation process from GRILLO-Werke with the intention of implementing it on an industrial scale. A Life Cycle Assessment (LCA) analysis demonstrated that the greenhouse gas (GHG) emissions of the new process are diminished by 85% in comparison to the conventional BASF process.^9^ The new process also outperforms the conventional one in other LCA impact categories. The primary factor contributing to these reductions is the capability to activate methane, which facilitates a one-step reaction for the synthesis of MSA, thereby significantly decreasing the complexity of the process. As previously mentioned, the conventional method initially necessitates the production of syngas (a CO/H_2_ mixture) and methanol, in addition to numerous supplementary reactions, all of which demand energy input and consequently generate a substantial quantity of indirect emissions and environmental impacts. An added benefit of the direct process is the utilization of reactants that are higher up in the value chain (methane and sulfur trioxide), which again necessitates less energy. The LCA study suggests that the sustainability of MSA production *via* the direct methane sulfonation process could be further enhanced by substituting natural gas, a non-renewable feedstock, with methane derived from biomass (biomethane extracted from biogas).

## Properties

### Physical properties

**Fig. 3:**
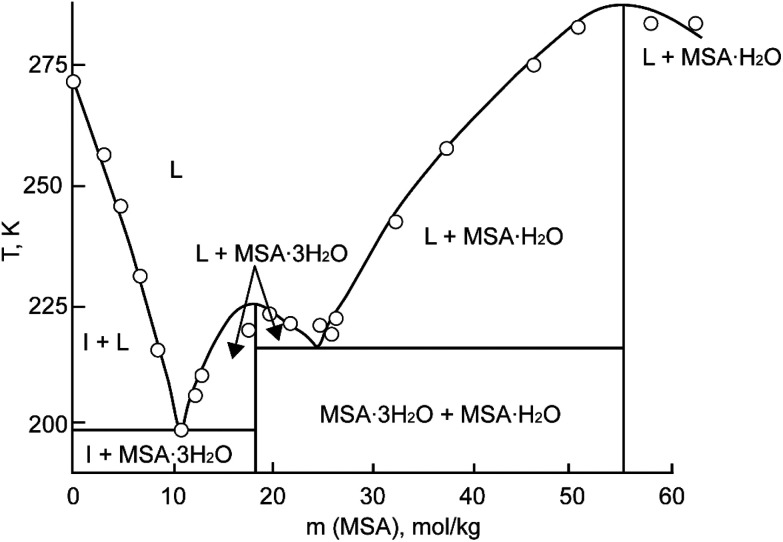
Part of the phase diagram of the MSA–water binary system (HMS = MSA). Redrawn from ref. 23 with permission from Springer Nature, copyright 2023.

MSA is commercially obtainable in two forms: an anhydrous form with a purity exceeding 99.5%, and as a concentrated aqueous solution with a weight percentage of 70%. In its anhydrous state, MSA manifests as a colorless, odorless, viscous liquid at room temperature. It exhibits a melting point of 19–20 °C.^3^ The 70 wt% aqueous solution melts at −54 °C. Part of the MSA–water binary phase diagram is shown in Fig. 3.^23^ Two crystal hydrates have been identified at low temperatures: the monohydrate CH_3_SO_3_H·H_2_O and the trihydrate CH_3_SO_3_H·3H_2_O. The monohydrate exists in a much larger temperature range (215.15–283.15 K) than the trihydrate, and it is expected that it can precipitate in applications involving an aqueous MSA solution at temperatures well below room temperature.

At atmospheric pressure, MSA decomposes above 185 °C before the boiling point is reached. MSA can be distilled without decomposition only at reduced pressures. Its boiling point is 167–167.5 °C at a pressure of 10 mmHg (13.33 hPa),^2^ and 122 °C at 1 mmHg (1.33 hPa). Because of its high boiling point, the vapor pressure of anhydrous MSA at room temperature is very low: 0.001 hPa at 23 °C.^24^ MSA is miscible in all proportions with water, ethanol, DMSO and THF. It is partially miscible with toluene and *n*-hexane. Table 1 gives an overview of the physicochemical properties of MSA.

**Table 1:** Summary of physicochemical properties of pure MSA^25,26^

Property	Value
Molecular weight [g mol^−1^]	96.1
Melting point [°C]	19
Boiling point [°C] at 13.3 hPa	167
Flash point [°C]	189
Autoignition temperature [°C]	>550
Mass density [g cm^−3^] at 25 °C	1.475
Vapor pressure [hPa] at 20 °C	0.013
Partition coefficient octanol/water, log *P*	−4.98
p*K*_a_ [—]	−1.92
Kinematic viscosity [m^2^ s^−1^] at 25 °C	7.6 × 10^−6^
Dynamic viscosity [mPa s] at 25 °C	11.21
Electrical conductivity [S cm^−1^] at 25 °C	0.59 × 10^−3^

### Acidity

The acid strength or acidity of a Brønsted acid HA in water is defined by the position of the following equilibrium:

10 HA + H_2_O ⇄ A^−^ + H_3_O^+^

The acid dissociation constant *K*_a_ is a quantitative measure for this acid strength and is defined as the equilibrium constant of eqn (10):11
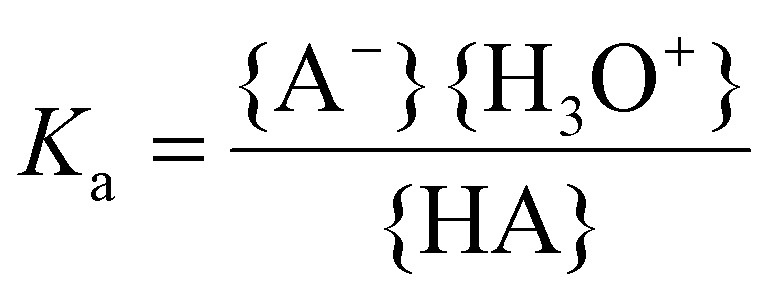


Here the brackets { } represent the activities of the species involved. For low concentrations (≪1 M), the activities might be approximated by the molar concentrations. Often the p*K*_a_ is reported instead of the *K*_a_ value:

12 p*K*_a_ = −log *K*_a_

Since a 0.1 M solution of MSA is more than 99.8% ionized, a precise determination of the activities or concentrations of the components at equilibrium is challenging. Covington and Thompson studied the ionization of MSA and other simple alkanesulfonic acids by Raman and ^1^H NMR spectroscopy and found a *K*_a_ value of 83 ± 2 for MSA at room temperature.^27^ This value corresponds to p*K*_a_ = −1.92. Having a p*K*_a_ value in the range −2 < p*K*_a_ <+2, the authors labeled MSA as a ‘moderately strong’ acid. In Table 2, the p*K*_a_ value of MSA in water is compared with those of other sulfonic and related acids (values rounded to one decimal).^28^ The table shows that MSA is the strongest acid of the series of *n*-alkanesulfonic acids, with general formula C_*n*_H_2*n*+1_SO_3_H. This can be explained by the electron-donating effect of the alkyl groups that counteracts the dissociation of the sulfonic acid group. The table also shows that MSA is a weaker acid than *p*-toluenesulfonic acid, which is MSA's competitor in several applications (*vide infra*). Being a weaker acid than sulfuric acid, MSA cannot be considered a super acid.

**Table 2:** p*K*_a_ values in water of sulfonic and related acids (at 25 °C)^28^

Acid	Formula	p*K*_a_
Propanesulfonic acid	C_3_H_7_SO_3_H	−1.5
Ethanesulfonic acid	C_2_H_5_SO_3_H	−1.7
**Methanesulfonic acid (MSA)**	**CH**_**3**_ **SO**_**3**_ **H**	−**1.9**
*p*-Toluenesulfonic acid (*p*TSA)	4-CH_3_C_6_H_4_SO_3_H	−2.7
Benzenesulfonic acid	C_6_H_5_SO_3_H	−2.8
Sulfuric acid	HOSO_3_H (H_2_SO_4_)	−3.0
Methoxysulfonic acid	CH_3_OSO_3_H	−3.5
4-Nitrotoluenesulfonic acid	4-O_2_NC_6_H_4_SO_3_H	−3.8
Trifluoromethanesulfonic acid (triflic acid)	CF_3_SO_3_H	−5.5
Fluorosulfuric acid (fluorosulfonic acid)	FSO_3_H	−5.6

### Chemical stability

MSA has a high chemical stability because of the low reactivity and the strength of the C–S bond, which resists normal hydrolysis under acidic or alkaline conditions. Methanesulfonate is remarkably stable against hydrolysis. When heated in an autoclave in the presence of an excess of a 3.7 M NaOH solution for 3 hours, no decomposition was detected at 315 °C.^29^ Under the same conditions, 1.5% decomposed at 345 °C and 11% at 375 °C.

**Fig. 4:**
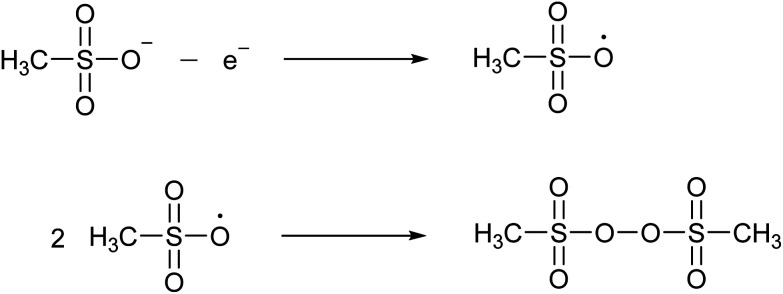
Formation of bis(methanesulfonyl)peroxide by anodic oxidation of the methanesulfonate anion to the methylsulfonyloxyl radical, followed by recombination of two radicals.

MSA is very resistant to strong oxidizing agents. For example, it does not react with hydrogen peroxide, nitric acid or potassium permanganate.^24^ It is described to be relatively stable in hot chromosulfuric acid (a mixture of CrO_3_ in concentrated H_2_SO_4_), although no quantitative data have been reported.^24^ MSA is also resistant to strong reducing agents, such as nascent hydrogen. MSA has a wide electrochemical window, extending on a platinum working electrode from about −2 V to +2 V *versus* the standard hydrogen electrode (SHE).^30^ At high anodic potentials (+2.9 to +3.7 V *versus* SHE) a concentrated MSA solution (10 M) undergoes an electrooxidation reaction. FTIR, Raman and NMR studies have indicated that the main oxidation product is bis(methanesulfonyl)peroxide, also called dimesylate peroxide. This is formed by recombination of two methylsulfonyloxyl radicals CH_3_S(O)_2_O˙ that originate from the anodic oxidation of the methanesulfonate anion (Fig. 4). Although this organic peroxide is stable at normal conditions, it can decompose explosively in air at temperatures above 70 °C.^31^

Because MSA is such a small molecule and a methyl group has a low reactivity, MSA does not undergo typical organic chemical reactions such as addition or substitution. MSA has no β hydrogen atoms, so that reactions such as β eliminations are excluded.

Upon heating, MSA decomposed in air before its boiling point is reached, but it is stable for a longer period of time to temperatures to 180 °C.^24^ Above 200 °C, thermal decomposition becomes faster and above 225 °C, MSA rapidly decomposes.^25^ To the best of our knowledge, no detailed thermal analysis studies have been performed on MSA yet, so the mechanism of thermal decomposition of MSA remains unknown.

### Corrosivity

While methanesulfonic acid (MSA) exhibits lower corrosivity in comparison to hydrochloric acid, a unique selling point, it would be erroneous to consider MSA a non-corrosive acid. Numerous metals utilized in construction materials can react with MSA, especially at elevated temperatures. Highly pure MSA demonstrates significantly less corrosivity than technical grades. An investigation involving three grades of austenitic stainless steel (304, 316, and 316Ti) in aqueous MSA solutions revealed that these stainless steel materials are highly passivated in the purest MSA solution and exhibit resistance against both uniform and localized corrosion.^32^ These stainless steels, commonly used in the mixing of reaction vessels, transportation, and storage vessels of MSA, are thus unaffected by pure MSA. However, MSA solutions containing higher amounts of impurities have been observed to induce corrosion issues. Chemical analysis identified chloromethanesulfonic acid in impure MSA, and it was proposed that this compound was responsible for the increased corrosion. In the context of tin electroplating baths, it has been noted that the MSA electrolyte is highly corrosive to the steel substrate, necessitating continuous removal of dissolved iron from the bath.^33^

### Biodegradability

Bacteria have also been isolated which can utilize MSA as a sole source of carbon and energy, whereby MSA is oxidized directly to formaldehyde and sulfite.^34^ These bacteria are specialized methylotrophs, all of which use a common enzyme for the initial cleavage of the C–S bond of MSA: *methanesulfonic acid monooxygenase*.^35^ The first isolated organism that was able to degrade MSA for growth, causing its complete mineralization, was the methylotroph strain M2. This bacterium is strictly aerobic and grows on several one-carbon (C1) substrates, including methanol, mono-, di- and tri-methylamine, formaldehyde and formate, as well as MSA. It is also capable of slower growth on other alkanesulfonates. MSA was oxidized only by bacteria previously grown on MSA, and not after growth on other C1 compounds. Suspensions of MSA-grown bacteria oxidized MSA completely to sulfate and carbon dioxide, with a stoichiometry of MSA : O_2_ = 1 : 2 and the production of increased acidity, consistent with the equation:^36^

13 CH_3_SO_3_^−^ + H^+^ + 2O_2_ → CO_2_ + H_2_O + SO_4_^2−^ + 2H^+^

It is postulated that a multitude of microorganisms, encompassing both aerobic and anaerobic types, are potentially capable of utilizing MSA, either as a substrate for growth or merely as a source of sulfur. However, these organisms have yet to be identified. Experimental data indicate that MSA is readily biodegradable in accordance with OECD guidelines 301A, 306, and 311, resulting in the formation of carbon dioxide, sulfate, water, and biomass.^37^ The oxygen demand for degradation is minimal due to MSA being a C1 compound. Given the absence of phosphorus, MSA does not contribute to eutrophication or the proliferation of algae.

## Metal methanesulfonates

### Preparation

Different methods for the preparation of methanesulfonate metals salts have been described in the literature. For detailed experimental procedures, the reader is referred to the 1999 paper of Gernon *et al.*^5^ The most straightforward method for producing a methanesulfonate metal salt involves the reaction of the acid with a stoichiometric amount of a metal oxide, hydroxide or carbonate. For instance, the reactions of silver(i) oxide, lithium hydroxide and lead(ii) carbonate with MSA are:

14 Ag_2_O + 2CH_3_SO_3_H → 2Ag(CH_3_SO_3_) + H_2_O

15 LiOH + CH_3_SO_3_H → Li(CH_3_SO_3_) + H_2_O

16 PbCO_3_ + 2CH_3_SO_3_H → Pb(CH_3_SO_3_)_2_ + CO_2_ + H_2_O

Although these reactions are formally equilibrium reactions, the equilibrium is shifted so far to the right that the reactions can be considered as essentially complete. The methanesulfonate salt can be recovered from the solution by evaporation of water *in vacuo* (4 mmHg). After crystallization of the hydrated salt, it is often washed with a volatile polar organic solvent such as ethanol, isopropanol or acetone, and dried to constant weight *in vacuo* (1 mmHg). Often, a slight excess of either MSA or the metal precursor is used. In instances where an excess of MSA is utilized, it is imperative to ensure the removal of the surplus acid from the final product. This can be accomplished by recrystallizing the metal salts from an appropriate solvent, most commonly water. When an excess of an insoluble metal precursor is employed, the precursor can be slurried in the MSA solution during the reaction, and the unreacted solid can be separated from the solution *via* filtration. If an excess of the soluble metal precursor is used, the metal methanesulfonate salt must be purified by recrystallization from water. Theoretically, the advantage of this method is the absence of unwanted anions introduced into the solution. Despite these reactions appearing straightforward, they are often slow; the surfaces of oxides and hydroxides tend to become passivated over time. Consequently, it is advised to conduct the reactions with freshly precipitated hydroxides or freshly calcined oxides. It is crucial to use pure precursors to prevent contamination of the solution. When technical grade oxides, hydroxides, or carbonates are used, it is frequently observed that an insoluble residue remains, even in the absence of an excess of precursor.

An alternative method for synthesis of silver(i) methanesulfonate is by reaction between AgNO_3_ and MSA, where the HNO_3_ formed is removed by distillation:^38^

17 AgNO_3_ + CH_3_SO_3_H → Ag(CH_3_SO_3_) + HNO_3_

Silver(i) methanesulfonate is a useful intermediate for conversion of metal chlorides into methanesulfonates. The driving force is the precipitation of silver(i) chloride. For instance, tin(ii) methanesulfonate has been prepared *via* this route:

18 SnCl_2_ + 2Ag(CH_3_SO_3_) → Sn(CH_3_SO_3_)_2_ + 2AgCl↓

An alternative approach for the synthesis of methanesulfonate salts from metal chloride salts involves a reaction between the metal chloride and MSA, succeeded by the distillation of hydrochloric acid or its removal *via* solvent extraction. A challenge associated with this method is the difficulty in completely eliminating all chloride ions, or in fully removing all organic solvents if solvent extraction is employed for the removal of hydrochloric acid.^39^

The technically and economically most attractive method to get access to methanesulfonate salts is by direct dissolution of metal in an aqueous MSA solution.^39^ In theory, this approach yields methanesulfonate salts of the highest purity, as long as pure metals are employed for dissolution. It is advisable to utilize fine metal powder to ensure an adequate surface area for acceptable reaction durations. This method is frequently utilized in the preparation of electrolyte baths for electroplating. The methanesulfonate salts of the more reactive metals can be produced through a direct reaction between the metal and MSA. For example, zinc metal reacts with MSA, resulting in the formation of zinc(ii) methanesulfonate and hydrogen gas:

19 Zn + 2CH_3_SO_3_H → Zn(CH_3_SO_3_)_2_ + H_2_

In a similar way, iron(ii) methanesulfonate is formed by reaction between metallic iron and MSA:

20 Fe + 2CH_3_SO_3_H → Fe(CH_3_SO_3_)_2_ + H_2_

The less reactive metals, and especially the precious metals, cannot be dissolved directly in MSA and the addition of an oxidizing reagent is required. The oxidizing reagent most frequently utilized is hydrogen peroxide, due to its availability in high purity and its inability to introduce impurities. For example, silver metal can be dissolved in MSA and transformed into silver(i) methanesulfonate in the presence of hydrogen peroxide, as demonstrated in the subsequent reaction:^40^

21 2Ag + 2CH_3_SO_3_H + H_2_O_2_ → 2Ag(CH_3_SO_3_) + 2H_2_O

Pure methanesulfonate salts can be prepared by anodic dissolution of the corresponding metal in a divided electrochemical cell. A divided cell is used to avoid the redeposition of the metal at the cathode, provided the metal can be electrodeposited from aqueous electrolytes. The electrochemical cell can be divided by an anion exchange membrane in an anolyte and catholyte chamber. This anion-exchange membrane allows transportation of methanesulfonate anions from the catholyte into the anolyte, but it prevents the transfer of metal cations from the anolyte into the catholyte. Anodic dissolution has been used to prepare the methanesulfonate salts of silver, palladium and lead, but it can be applied to other metals as well.

When metal methanesulfonate salts are prepared by dissolution of the metal in MSA in the presence of an oxidizing reagent or by anodic dissolution of the metal, it is crucial to meticulously select the process variables. This is to prevent the formation of undesired oxidation states of the metal, such as tin(iv) instead of the desired tin(ii). Theoretically, there is also the potential risk of oxidative decomposition of MSA leading to sulfate anions or the generation of malodorous sulfur-containing reduction products. While literature describes MSA as a redox-stable molecule, there are limited studies available concerning the electrochemical decomposition reactions of MSA.

### Solubilities

Metal methanesulfonate salts are very soluble in water (Table 3); at room temperature their solubilities are often in the order of hundreds of grams per liter. Methanesulfonate salts share this property with the nitrate salts, although the methanesulfonate anion is (electro)chemically much more stable than the nitrate ion. When a metal ion forms poorly soluble sulfate and/or chloride salts, a highly concentrated solution of that metal ion can be obtained by dissolving the corresponding metal methanesulfonate salt. This is particularly applicable to lead(ii), silver(i), calcium(ii), strontium(ii), and barium(ii). Additionally, for tin(ii), mercury(ii), silver(i), and the trivalent rare-earth ions, the solubilities of the methanesulfonates markedly exceed those of the corresponding sulfates.^5,24^ For instance, the solubility of Nd(CH_3_SO_3_)_3_ in water is 1069 g L^−1^ at 23 °C,^24^ whereas the solubility of Nd_2_(SO_4_)_3_·8H_2_O is only 80 g L^−1^ at 20 °C.^41^ However, for metal ions that do form well-soluble sulfate and/or chloride salts, the metal concentration in the saturated methanesulfonate solution is sometimes even lower than that of the saturated sulfate or chloride solutions. For instance, the Zn^2+^ concentration in a saturated Zn(CH_3_SO_3_)_2_ solution is 2.16 mol L^−1^, whereas in concentrated ZnSO_4_ and ZnCl_2_ solutions it is 3.32 and 13.0 mol L^−1^, respectively.

**Table 3:** Metal concentrations in saturated aqueous solutions of selected methanesulfonate salts, compared with those of the corresponding sulfate and chloride salt (at 22 °C). Metal concentrations are given in gram per liter (g L^−1^), followed by the corresponding molar concentration (mol L^−1^). Adapted from ref. 5

Metal ion	Methanesulfonate	Sulfate	Chloride
Li^+^	49 (7.06)	34 (4.90)	65 (9.37)
Na^+^	130 (5.65)	64 (2.78)	128 (5.57)
K^+^	175 (4.48)	49 (1.25)	151 (3.86)
Mg^2+^	34 (1.40)	64 (2.63)	122 (5.02)
Ca^2+^	117 (2.92)	1.0 (0.0249)	221 (5.51)
Sr^2+^	223 (2.55)	0.0 (0.00)	266 (3.04)
Ba^2+^	218 (1.59)	0.0 (0.00)	235 (1.71)
Mn^2+^	159 (2.90)	193 (3.52)	226 (4.12)
Co^2+^	149 (2.53)	127 (2.16)	228 (3.87)
Ni^2+^	125 (2.13)	143 (2.44)	257 (4.38)
Cu^2+^	127 (2.00)	86 (1.35)	309 (4.87)
Ag^+^	401 (3.72)	6.0 (0.0556)	0.0 (0.00)
Zn^2+^	141 (2.16)	217 (3.32)	850 (13.0)
Cd^2+^	360 (3.20)	348 (3.10)	641.86 (5.71)
Sn^2+^	443 (3.73)	168 (1.42)	583 (4.91)
Hg^2+^	363 (1.81)	0.0 (0.00)	48 (0.239)
Pb^2+^	539 (2.60)	0.0 (0.00)	7.0 (0.0338)

When comparing different literature sources, we must carefully check whether the solubilities are expressed in metal concentrations in the saturated solution, or as the solubility of the metal salt. For instance, for Pb^2+^, an aqueous saturation solubility of 2.60 mol L^−1^ is provided in ref. 5, which corresponds to a metal concentration of 539 g L^−1^, whereas in ref. 37 a value of 1075 g L^−1^ is mentioned, which refers to the solubility of the Pb(CH_3_SO_3_)_2_·H_2_O salt (MW = 415.41 g mol^−1^).

Compared to other metal salts, very few detailed data have been reported in the literature on the solubility of methanesulfonate salts in water. For most metal ions, experimental data are available only at room temperature. Sparse data are available on the temperature dependence of the metal methanesulfonates, whereas it is known that highly soluble metal salts often show a strong temperature dependence of their solubility. Related to the solubilities in aqueous solutions are the phase diagrams showing solid–liquid equilibria. The solid–liquid equilibria of the Zn(CH_3_SO_3_)_2_·H_2_O and Cu(CH_3_SO_3_)_2_·H_2_O binary systems have been investigated.^42–44^ Similar studies have been performed on the Ni(CH_3_SO_3_)_2_·H_2_O, Co(CH_3_SO_3_)_2_·H_2_O and Mn(CH_3_SO_3_)_2_·H_2_O binary systems.^45^ An important insight obtained from these studies is that the methanesulfonate salts of the transition metal ions have the tendency to form different crystal hydrates (crystalline compounds with several water molecules in the crystal structure). Higher hydrates (*i.e.*, with a larger number of water molecules per metal ion) are formed at temperatures below room temperature. These higher hydrates have much lower solubilities than the lower hydrates. As a consequence, the solubility of the methanesulfonate salts can show a steep decrease when solutions are being cooled below room temperature. These studies on hydrate formation also indicate how important it is for solubility studies to report which solid phase is in equilibrium with the solution. If the solubility measurements are carried out too fast, there is the possibility that metastable solid compounds are formed, with a solubility different from that of the thermodynamically stable phases. Short equilibration times can lead to erroneous solubility data.^46,47^ The phase behavior of the Mg(CH_3_SO_3_)_2_·H_2_O binary system has been investigated and it was observed that Mg(CH_3_SO_3_)_2_·12H_2_O crystallized out at lower temperatures.^48^ This shows that also several alkaline earth metal methanesulfonates have a tendency to form higher hydrates upon cooling.

It is evident that further research on the solubility of metal methanesulfonates is required before these systems can be fully understood. An enormous amount of experimental thermodynamics data as a function of temperature and pressure are available for aqueous electrolytes containing chloride, sulfate and/or nitrate salts. On the contrary, methanesulfonate salts are largely absent in compilations of thermodynamic data for aqueous electrolytes.^49^

Although the metal methanesulfonate salts are highly soluble in water, addition of MSA to the water will drastically reduce the solubility of these metal methanesulfonates. The negative effect of addition of an acid on the solubility of its metal salts is well known for other metal salts and it often explained by the common-ion effect (Le Châtelier principle). However, at high MSA concentrations, an additional effect must be taken into account: so many water molecules will be protonated that the amount of free water, and hence the water activity, will be significantly lowered. In other words: there are not enough free water molecules left to fully saturate the first coordination sphere of the metal cations by hydration. This reduction of solubility in the presence of MSA has been reported for silver(i),^50^ copper(ii),^42^ and zinc(ii) methanesulfonates.^42^ This decrease in solubility in aqueous solutions is a factor that must be taken into account when preparing electrolytes for electrodeposition. In contrast to their high solubility in aqueous solution, metal methanesulfonates are poorly soluble in anhydrous MSA, especially at room temperature. This is evident when anhydrous MSA is being used to leach or dissolve solid materials; precipitates are often formed upon cooling the solution.^51,52^

**Fig. 5:**
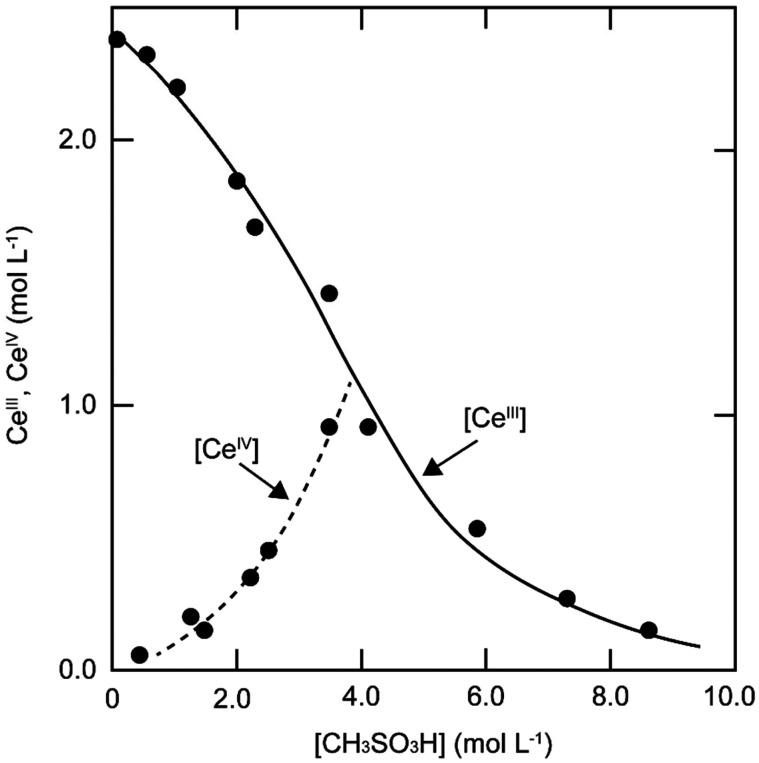
Solubility of cerium(iv) and cerium(iii) methanesulfonate in aqueous solutions as a function of the MSA concentration. Redrawn from ref. 53 with permission from Springer Nature, copyright 1990.

**Fig. 6:**
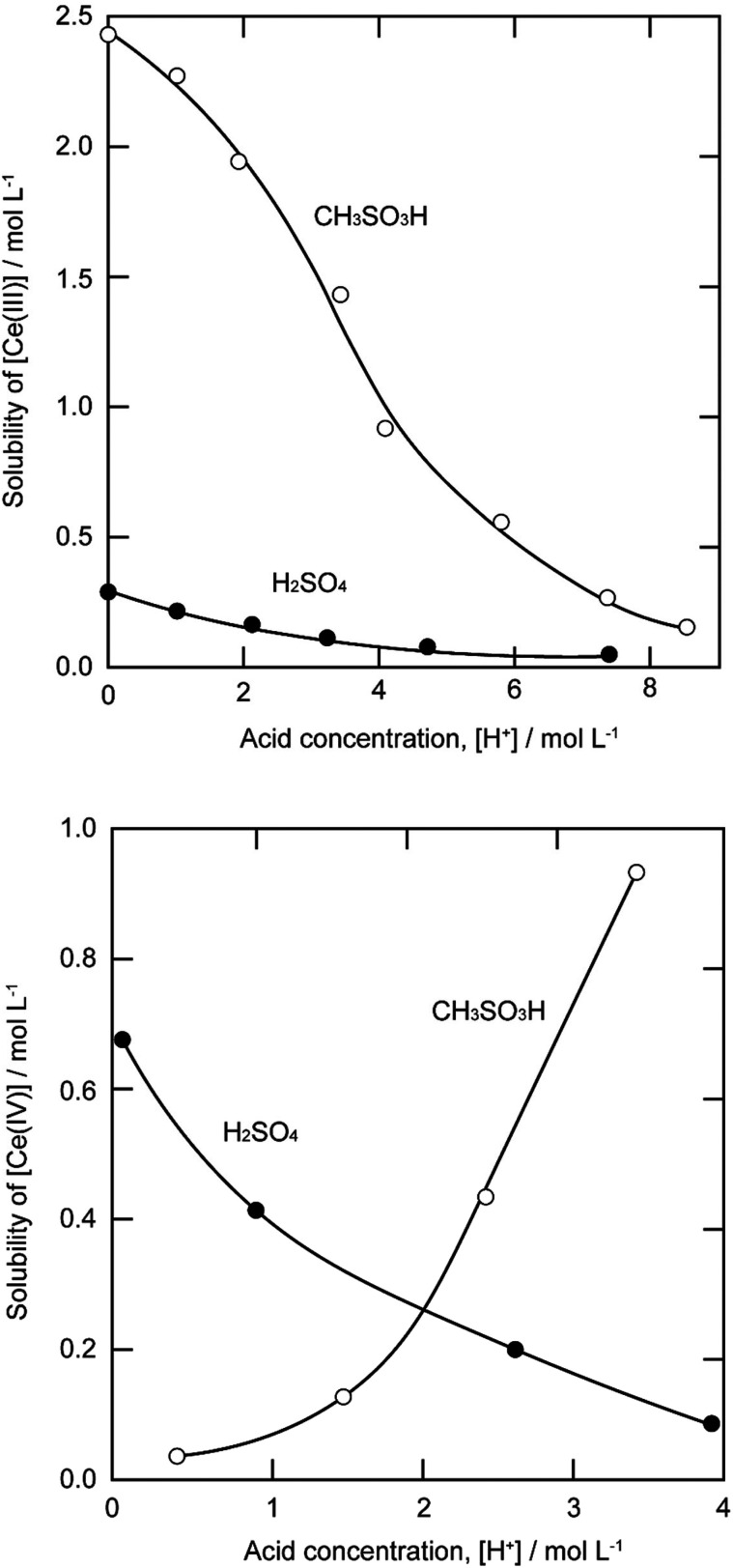
Comparison of the solubility of cerium(iii) (top) and cerium(iv) ions (bottom) in aqueous methanesulfonic and sulfuric acid electrolytes at 22.5 °C. Redrawn from ref. 54 with permission from Elsevier, copyright 2012.

The solubility of cerium(iii) and cerium(iv) methanesulfonates has been investigated in more detail than the solubility of other methanesulfonate salts, because of the use of these salts as electrolytes in redox flow batteries (*vide infra*). Cerium(iii) and cerium(iv) methanesulfonate show opposite trends in their solubility as a function of the MSA concentration: the solubility of cerium(iii) methanesulfonate decreases with increasing MSA concentration (as expected), but the solubility of cerium(iv) methanesulfonate increases with an increase in MSA concentration (Fig. 5).^53^ At low MSA concentrations, cerium(iv) methanesulfonate is poorly soluble in contrast to most other methanesulfonate salts: only about 0.1 mol L^−1^ cerium(iv) dissolves in 1 M MSA and the salt is even hardly soluble when no MSA is added. The solubility of cerium(iv) increases sharply with increasing acid concentration (about 1 mol L^−1^ cerium(iv) dissolves in a 4 M MSA solution). The limited solubility of cerium(iv) methanesulfonate at low MSA concentrations is probably due to hydrolysis of cerium(iv) and formation of poorly soluble basic cerium(iv) salts. It was observed that a yellow powder with composition Ce(CH_3_SO_3_)(OH)_2_·H_2_O precipitated from dilute cerium(iv) methanesulfonate solutions. The solubilities of cerium(iii) and cerium(iv) in MSA and sulfuric acid solutions as a function of the proton concentration are compared in Fig. 6.^54^

Upon examining the solubility data for methanesulfonate and sulfate salts, it may appear that using methanesulfonate salts offers little benefit for most metal ions. However, there are exceptions, including lead(ii), tin(ii), silver(i), mercury(i), calcium(ii), strontium(ii), barium(ii), and trivalent rare-earth ions. For these ions, the methanesulfonate salts exhibit a (much) higher solubility than their sulfate counterparts. It has been mentioned that addition of acid decreases the solubility of salts, but this is valid for both MSA and sulfuric acid. But it is important to understand that the solubility data reported thus far pertains to single metal salts and the solubility differences between methanesulfonate and sulfate salts can be much more significant in certain cases. Double salt formation is very common in sulfate systems and can lower the solubility of the metal ion in solution. For instance, addition of a solution of Na_2_SO_4_ to a solution of trivalent rare-earth sulfates leads to the formation of poorly soluble sodium rare-earth double sulfates.^55^ For transition metal sulfates, the tendency to form poorly soluble double salt is pronounced in the presence of potassium and ammonium sulfate. To the best of our knowledge, metal methanesulfonate salts seem to have much less tendency to form double salts, although further research must confirm this statement.

All the solubility data for methanesulfonate salts reported in the literature are solubilities in water. As far as we know, no solubility data are available for organic solvents nor for mixtures of water and polar organic solvents. Such information could be very helpful for the development of solvometallurgical processes,^56^ or antisolvent crystallization of methanesulfonate salts.^57^

The above-mentioned steep decrease of the solubility of metal methanesulfonates upon cooling when higher hydrates are formed can be exploited to recover metal methanesulfonates from solution. For instance, Gernon *et al.* describe how iron impurities can be removed from a tin(ii) methanesulfonate electrolyte for tin electroplating by cooling of the contaminated solution, leading to precipitation of iron(ii) methanesulfonate.^5,58^

### Complex formation in solution

The literature presents scant information on the solution chemistry of methanesulfonate salts. The methanesulfonate anion is a weakly coordinating anion so that it can be assumed that in diluted aqueous solutions it resides only in the second coordination sphere, which means that the metal ions have only water ions in the first coordination sphere and there is no direct interaction between the metal cation and the methanesulfonate anion. However, in more concentrated solutions, it is possible that metal–methanesulfonate bonds can be formed. Such bonding has been observed in the crystal structures of metal methanesulfonate hydrate salts.^45^

The formation of metal complexes between lead(ii) and methanesulfonate ions has been studied by polarography on a dropping mercury electrode.^59^ The authors describe the stepwise formation of the complexes [Pb(CH_3_SO_3_)]^+^, [Pb(CH_3_SO_3_)_2_] and [Pb(CH_3_SO_3_)_3_]^−^ and determined the overall stability constants *β*_*i*_ (*i* = 3). These values are very small, in agreement with the weak tendency of the methanesulfonate ion to form complexes in aqueous solution. The authors state that at MSA concentrations above 1.8 M, the [Pb(CH_3_SO_3_)_3_]^−^ complex dominates. However, one must be cautious with the interpretation of these results, because there is no experimental evidence for the formation of inner-sphere complexes (*i.e.*, the methanesulfonate ions in the first coordination sphere). There is obviously a need for more detailed studies on the speciation of metal methanesulfonate complexes in concentrated aqueous solutions and the measurement of the corresponding complex formation constants.

## Reagent, catalyst and solvent in organic synthesis

MSA has been tested as a reagent in organic synthesis, especially for acid-catalyzed reactions. Here, MSA is used in concentrated form and not as a 70 wt% aqueous solution. MSA is utilized in the first place as a superior alternative to concentrated sulfuric acid. Because concentrated sulfuric acid is not only a strong Brønsted acid, but also a dehydrating, oxidizing and sulfonating agent, the use of sulfuric acid in organic synthesis is often accompanied by side reactions and decomposition reactions. Sulfuric acid leads to coking (formation of charcoal), the formation of resinous and gum-like materials. The reaction mixtures are frequently darkly colored by dye-like decomposition products. Hydrochloric acid suffers from high corrosivity so that no stainless steel reactors can be used, and often toxic chlorinated side products are formed. For instance, alcohols can be transformed into chloroalkanes. These issues are not encountered when MSA is used instead of sulfuric or hydrochloric acid, although in principle concentrated MSA can cause dehydration reactions at high temperatures. In general, the use of MSA leads to higher yields, higher selectivities and purer final products.

For these organic reactions, *p*-toluenesulfonic acid (PTSA) could also be used instead of MSA. PTSA and MSA have in many aspects similar properties, but PTSA is a solid at room temperature, whereas MSA is a liquid. Although a solid might seems to be more convenient for organic reactions at the laboratory scale, liquids are much easier to handle at an industrial scale. Addition of a liquid rather than a solid to a chemical reactor is way more convenient.

### Esterification reactions

In acid-catalyzed esterification reactions (Fischer esterification), an alcohol is reacted with a carboxylic acid in the presence of a strong Brønsted acid, to form an ester and water:^60^

22 RCOOH + R′OH ⇄ RCOOR′ + H_2_O

The equilibrium of this reaction is shifted to the direction of ester formation by removal of the ester and/or the water. The function of the acid is to protonate the carbonyl group of the carboxylic acid to make it more susceptible to nucleophilic attack by the alcohol. Traditionally concentrated sulfuric acid was used as the acid catalysts for this reaction, because it combines a high acid strength with dehydrating abilities. However, the strong dehydrating power can also lead to coking and the dehydration can trigger unwanted side reactions such as ether formation. The use of MSA can avoid issues with coking, gum formation, formation of resinous materials, or formation of dehydration side products.^4^ Generally, the production yields of ester formation are superior with MSA compared to sulfuric acid when used as a catalyst. An additional benefit of MSA is the reduced formation of colored impurities, resulting in esters of higher purity and quality. This is particularly crucial for esters utilized in industries such as food production, cosmetics, and pharmaceuticals.

**Fig. 7:**
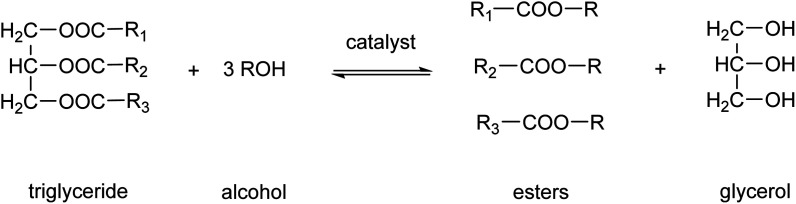
Transesterification reaction to transform a triglyceride into monoalkyl esters and glycerol.

Biodiesel production is one of the main applications of MSA in esterification reactions.^61,62^ Biodiesel is a renewable fuel comprised of monoalkyl esters of long-chain fatty acids.^63^ The feedstocks used to produce biodiesel are known as triglycerides. Sources of these triglycerides are soybeans, rapeseeds, coconuts, recycled cooking oil or animal fats. Biodiesel is produced by a chemical process known as *transesterification*, by which the triglycerides are reacted with alcohols, in the presence of a catalyst, to produce fatty acid alkyl esters (Fig. 7).^64^ The predominantly used alcohol for this reaction is methanol, leading to formation of methyl esters. Glycerol, also known as glycerine, is the main byproduct of this biodiesel production. Acids or bases can be used as catalyst for the transesterification reaction. MSA can replace more traditional acid catalysts such as sulfuric acid or hydrochloric acid in the acid-catalyzed transesterification.^65^ At the elevated temperatures necessitated for esterification with sulfuric acid, the sulfuric acid not only catalyzes the esterification reaction but also functions as a sulfonating and dehydrating agent. This results in the partial sulfonation of the fatty acids and the formation of coke *via* dehydration. The challenges associated with the use of hydrochloric acid include its corrosive nature and the generation of chloromethane as an undesired byproduct. The employment of MSA enables the mitigation of these issues. Furthermore, MSA also contributes to a reduction in the discoloration of the biodiesel. In instances where the catalyst is a base, such as NaOH or KOH, MSA can be utilized for the neutralization of the basic catalysts following transesterification.^66^ It was found that the use of MSA led to much higher yields of monoalkyl esters than when sulfuric acid was used for the neutralization reaction.

### Alkylation reactions

**Fig. 8:**
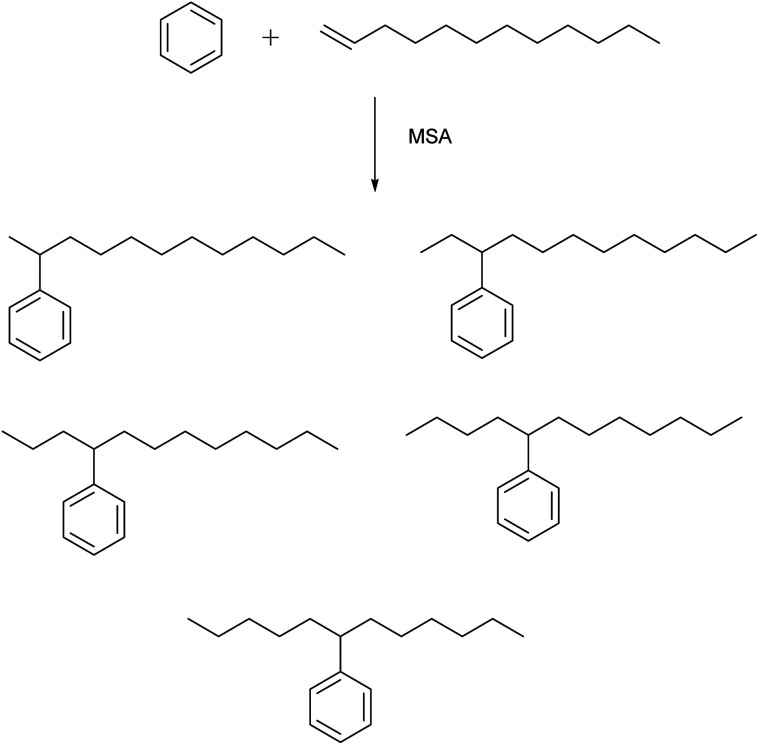
Isomers that can be formed in the alkylation of benzene with 1-dodecene, catalyzed by MSA.

MSA is an active catalyst for alkylation reactions. An example is the electrophilic addition of long-chain olefins such as 1-dodecene to benzene.^67^ Alkylbenzenes with long linear alkyl chains are useful intermediates for the production of anionic surfactants such as sodium dodecylbenzenesulfonate. In the alkylation of benzene with 1-dodecene, the desired products are the monoalkylated compounds, particularly 2-phenyldodecane because of its better emulsibility and its good biodegradability. During this alkylation reaction, five different isomers of the monoalkylated product, 2- to 6-phenyldodecane, can be formed, because of a shift of the charge in the carbenium intermediate (Fig. 8). The 1-phenyl isomer is not formed, as the reaction follows the Markownikow rule. Traditionally, HF or AlCl_3_ are used as catalysts for these Friedel–Crafts alkylation reactions. However, HF is highly toxic, volatile, and corrosive; AlCl_3_ is also corrosive, and it must be destroyed by hydrolysis in the working-up procedure. This leads to formation of corrosive HCl gas. High selectivities in excess of 90% for monoalkylated phenyldodecanes and conversion yields of 98% for 1-dodecene were observed. The alkylation reaction of aromatics with MSA as catalysts has also been applied for removal of traces of alkenes (olefins) from aromatics in the petroleum processing.^68^

### Fries rearrangement

**Fig. 9:**
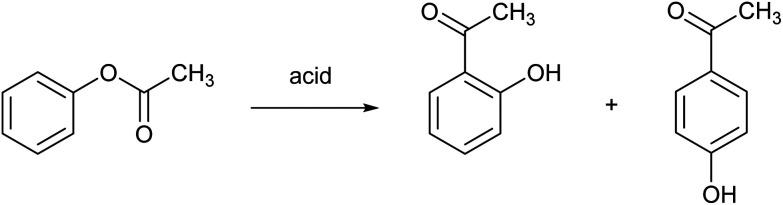
Fries rearrangement reaction of phenyl acetate.

The Fries rearrangement reaction is the conversion of phenolic esters into hydroxyaryl ketones on heating in the presence of a Brønsted or a Lewis acid catalyst. It involves migration of the acyl group of an phenol ester to the aryl ring. It is an *ortho*, *para*-selective reaction, and is used in the preparation of acyl phenols. An example is the Fries rearrangement of phenyl acetate to a mixture of *ortho*-hydroxyacetophenone and *para*-hydroxyacetophenone and (Fig. 9). This reaction is the first step of the Hoechst Celanese manufacturing process of the analgesic drug paracetamol. Currently used catalysts are HF, BF_3_ or AlCl_3_, and each of these have their safety and environmental issues as explained elsewhere in this review. MSA was found to be a very efficient catalyst in the Fries rearrangement of phenyl acetate, with quasi-quantitative conversion yields and excellent selectivities to the *para* product (the desired product) at an isomer ratio *para*/*ortho* of about 10.^69^ It was found that anhydrous MSA must be used, as traces of water immediately hydrolyzed phenyl acetate to phenol and acetic acid. Although MSA is called a catalyst, large amounts of MSA are required to obtain high conversion yields: a molar ratio MSA to phenyl acetate of 8 : 1 gave the optimum result for a reaction at 90 °C.

### Solvent for polymers

**Fig. 10:**
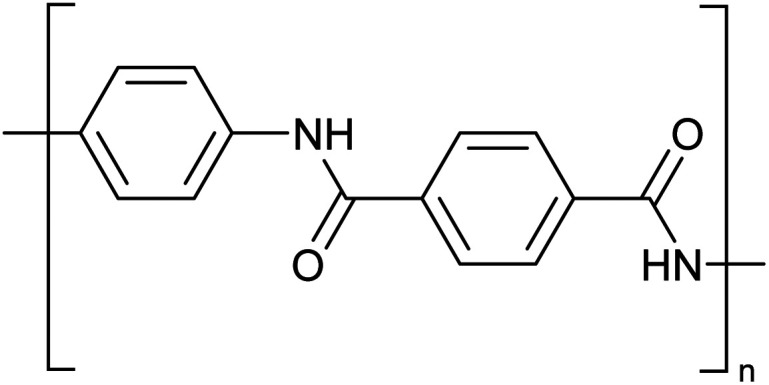
Structure of poly-*p*-phenylene terephthalamide (PPTA).

Concentrated MSA can replace concentrated sulfuric acid as a solvent for dissolution and processing of poorly soluble polymers such as polyaramids. A typical example of a polyaramid polymer is poly-*p*-phenylene terephthalamide (PPTA) (Fig. 10).^70^ This *para*-aramid is obtained by low-temperature polycondensation of *p*-phenylenediamine and terephthaloyl chloride, with release of HCl.^71^ Through the formation of an intermolecular hydrogen bond network, the PPTA chains self-organize into larger “sheets”. In turn, these sheets stack on top of each other *via* π–π-interactions, forming larger fibrillar structures. All the PPTA chains are aligned more or less parallel to each other, and as a result the fibrils are very strong along their axis. This supramolecular structure makes PPTA ideal for the production of high tensile strength fibers, such as Kevlar® or Twaron®. These fibers have very high tensile strengths while still being light-weight, resulting in a strength-to-mass ratio that is 5 times higher than that of steel. While originally developed to replace steel wires as tire reinforcement, PPTA fibers now serve in a broad range of applications such as ropes and cables, sports equipment, musical instruments, cut-resistant clothing, ballistic protection, composite materials and many more.

As polymer solutions become more viscous with increasing polymer chain length, a higher inherent viscosity indicates a longer polymer chain. PPTA is generally processed by spinning it into fibers. The PPTA powder is dissolved in concentrated sulfuric acid at a concentration of about 20 wt%, and PPTA is spun using a technique called ‘dry jet wet spinning’. Although PPTA is chemically quite a stable molecule, the molecule can undergo hydrolysis when dissolved in concentrated sulfuric acid.^72^ This hydrolysis will lead to a lower inherent viscosity, indicating that the average molar mass of the polymer chains decreases. Concentrated sulfuric acid is known to be a sulfonating agent so that there is the risk that the phenyl groups in PPTA will be sulfonated. Moreover, concentrated sulfuric acid is an oxidizing acid so that unwanted oxidation reactions can occur as well. To mitigate the adverse properties of concentrated sulfuric acid, concentrated MSA has been explored as solvent for PPTA and related polymers such as poly(*p*-phenylene-*cis*-benzobisoxazole) (PBO).^73–75^ However, these studies do not reveal a distinctive advantage of MSA over sulfuric acid.

MSA has also been applied as solvent for the processing of poly(ether-ether-ketone) (PEEK) for the fabrication of solvent-resistant membranes for nanofiltration.^76^ PEEK is a semi-crystalline polymer with very low fractional free volume, an exceptional solvent resistance and a high thermal stability. It is insoluble in conventional organic solvents, but can be dissolved in concentrated MSA or sulfuric acid, or mixtures thereof.

## Descaling and cleaning applications

MSA is widely used as a scale-removal chemical.^77^ Scale formation refers to the precipitation of sparingly soluble inorganic salts from an aqueous medium.^78^ It poses a significant challenge not only in the oil and gas sector, but also in the mining and metallurgical industries. Oilfield scales originate from the precipitation of water naturally present in reservoir rocks or when two incompatible waters encounter each other downhole, leading to oversaturation of the produced water with scale constituents. Scale deposition can transpire on any surface, and once initiated, it will progressively thicken over time unless some form of remedial action is undertaken. The formation of scale is detrimental to companies as it adversely affects operational efficiency. The accumulation of scale constricts pipes and tubing, impeding the flow of fluids. The emergence of scale in pipelines, tanks, heat exchangers, pumps, and sprayers results in diminished flows, escalated energy costs, reduced heating or cooling efficiency, and expensive downtime for cleaning and scale removal.

The most common types of scales include sulfates (Ba, Sr, Ca), oxides/hydroxides (Fe, Mg), carbonates (Ca, Mg, Fe), and sulfides (Fe). The scale deposition depends on several factors such as temperature, pressure, chemical reaction equilibria, pH, contact time, evaporation, and ionic strength.^79^ The scale deposits can be as a single mineral phase, but usually, scales are composed of a combination of different elements.

The most problematic scale in hydrometallurgical operations is gypsum (CaSO_4_·2H_2_O), because limestone, lime or slaked lime are used for neutralization of the pregnant leach solution after leaching of ores or concentrates with sulfuric acid. These bases react with the sulfuric acid to form a precipitate of gypsum, that is separated from the liquid by a solid/liquid separation method such as filtration or *countercurrent decantation* (CCD). The resulting liquid is saturated in calcium sulfate, and it is easy to generate a supersaturated solution when the solution is cooled somewhere else in the circuit. This can lead to unwanted crystallization of gypsum that adheres to the inner walls of tubes, pipes or reactors.

Although chemical scale inhibitors can be added to solutions, it is often not possible in hydrometallurgical operations, because these chemicals can have unwanted side effects. Therefore, periodic cleaning of the equipment to remove accumulated scale is often the preferred option. Although MSA can be used for removal of different types of scales, it is most suited for removal of carbonate and oxide scales. Carbonate scales, such as calcite, aragonite and vaterite (all CaCO_3_ polymorphs), siderite (FeCO_3_) and dolomite (CaMg(CO_3_)_2_) have a very high solubility in MSA and can effectively be removed using MSA. Among the oxides we have mainly brucite (Mg(OH)_2_) and periclase (MgO). For iron, different hydroxide and oxyhydroxide phases, such as goethite (FeOOH) are soluble in MSA. MSA can also be used to remove iron rust (*vide infra*). Sulfate scales such as barite (BaSO_4_), strontianite (SrSO_4_), anhydrite (CaSO_4_) and gypsum have very low acid solubility and cannot be dissolved in MSA. The use of some convertor such as Na_2_CO_3_ and NaOH can transform gypsum to acid-soluble compounds that can be removed using MSA. In general, convertors such as NaOH and Na_2_CO_3_ can used to convert several acid-insoluble scales into acid-soluble scales.

Calcium sulfate scale can be removed by MSA, but extended reaction times and heating is required. Less common scales that are difficult to remove and require elevated temperatures are calcium oxalate and calcium phosphate scales. Sulfide scales such as troilite (FeS) and pyrrotite (Fe_7_S_8_) are problematic because they react with MSA with formation of hydrogen sulfide (H_2_S) gas. Hydrogen sulfide gas is extremely toxic, heavier than air and its strong and distinctive smell disappears at lethal concentrations, as the olfactory receptors are saturated.

The primary application area for MSA is the safe and efficient removal of scale from pipelines, while minimizing corrosion of the pipelines themselves.^77^ Additionally, MSA can be applied to other areas, including descaling pumps, heat exchangers, tanks, and mixers, provided appropriate safety procedures are followed. MSA formulations may include other acids, corrosion inhibitors, oxidizing and reducing agents, chelating agents, and surfactants to optimize performance in descaling sensitive alloys. One significant advantage of MSA over hydrochloric acid, which is also commonly used for scale removal, is that MSA exhibits much lower corrosiveness. MSA is also much easier and safer to handle than hydrochloric and nitric acid. It releases no fumes and is odorless.

MSA can be found in formulations for removal of rust and rust stains from all types of surfaces.^80^ Another cleaning application is removal of limescale and soap scums for cleaning bathroom surfaces.^81^ In industrial facilities for conversion of renewable biobased feedstock in biochemicals, biomaterials, biofuels and other bioproducts by biomass pretreatment, biocatalysis, fermentation and green chemical reactions, as well as in food production facilities, diaries and breweries, the manufacturing equipment becomes soiled with carbohydrates, protein, fat, mineral and oil soils on their inner surfaces. Over time, these soils are transformed into hard-to-remove complex residues. MSA finds also application in *clean-in-place* (or CIP) techniques that can be used to remove soils from the internal components of tanks, lines, pumps and other processing equipment, without resorting to dismantling the equipment in order to removal difficult soils manually.^82^ This dismantling is expensive and time-consuming.

## Electroplating

Electroplating is an electrochemical process to cover the surface of a metal by a thin layer of another metal, mainly for corrosion protection or for decorative purposes. By electroplating it is possible to cover the substrate metal with a more uniform and thinner layer of the coating metal than by immersing the substrate metal into a molten metal bath. The possibility to use MSA-electrolytes for electroplating of different metals such as copper, zinc, cadmium, lead, nickel, and silver have been recognized for a long time,^4,83^ but MSA has been found to be very suitable for electroplating of lead, tin and lead–tin solder. In this application, it offers an environmentally friendlier alternative for electrolyte baths based on fluoroboric acid (HBF_4_) and fluorosilicic acid (H_2_SiF_6_).

Tin(ii) methanesulfonate in MSA is a widely used electrolyte for the preparation of tin-coated metals, especially tin-coated steel, known as *tinplate*.^84,85^ The tin layer on each surface is typically about 0.38 to 1.6 μm thick, whereas the tin-coated steel strip itself typically has a thickness between 0.15 and 0.60 mm. Cans made of tin-plated steel (tin cans) are widely used in packaging, such as the packaging of food and beverages, as well as in the packaging of other materials, such as paint of motor oil. In the high-speed tin-plating of steel strips, the strips of steel are first cleaned in an alkaline solution to remove oils and greases. Then, the steel strip passes through several water rinses and then into a dilute acid solution (*pickling solution*) before passing into the electrolyte plating bath, which deposits a layer of tin metal on the steel surface. The as-deposited layer of tin has a smooth, matte surface. After plating, the plated steel strip is typically rinsed twice with water. The purpose of the rinsing steps is to remove as much of the components of the plating electrolyte solution from the tin surface as possible, since some of the electrolyte solution is retained on the tin surface as *drag out* when the tinplate is removed from the plating bath. After rinsing, the plated strip enters a fluxing solution, followed by air drying. The term ‘*fluxing*’ refers to a substance that aids the reflow process. In the reflow process (also called reflowing, flow melting, or flow brightening), the plated strip is heated in an oven momentarily to a temperature slightly above the melting point of tin (232 °C), typically to 240 °C. During reflow, the tin layer is melted, forming a surface later of tin and a subsurface diffusion layer containing tin and tin–iron alloy on the steel substrate. After this heating step, the tin-plated strip is rapidly cooled or quenched by immersion in water, producing a tin surface layer that has a bright finish. In a continuous steel strip plating line, the steel strip travels at speeds from 2 to 10 m s^−1^, while the current density varies from 1000 to 6500 A m^−2^ depending on the steel grades and the coating masses.^86^ Furthermore, the stannous ion concentration is relatively low, in the range of 84 to 168 mM, to minimize the loss of costly chemicals through leaks and drag out in a continuous steel strip plating line.

The tin plating solution based on MSA and tin(ii) methanesulfonate is known as the RONASTAN® system, and was developed by the company LeaRonal (in 1999 sold to Rohm and Haas). In 1989, the first commercial tinplate production line using the RONASTAN® process was implemented by the steel company Koninklijke Hoogovens (now Tata Steel IJmuiden) in IJmuiden, The Netherlands. At present, this is the most widespread technology for producing tinplate.^87^ The advantages that are claimed include: high conductivity, biodegradability, low incidence of tin(iv) generation, wide current density range and adaptability of process attributes to end user requirements through the use of modern grain refiners.^88^

Tin electrodeposition under acidic conditions necessitates the incorporation of organic additives, given that tin coatings deposited without these additives in the electrolyte are non-adherent, coarse, porous, and prone to dendrite formation. These additives also enhance the throwing power of the solution. Organic additives can be categorized into surface active agents, oxidation inhibitors, grain refiners, and brighteners. The optimal additive will exhibit minimal decomposition under standard usage to reduce the occlusion of organics in the deposits. The presence of substantial amounts of occluded organics and rough deposits can lead to surface corrosion and issues with solderability. The chemical structure of the organic additives is highly diverse, encompassing aromatic sulfonates, amine derivatives, phenol derivatives, unsaturated carbonyl compounds, polyoxyethylene and polyoxypropylene, glue, gelatin, wood tar, resins, and others.^89^ The development of these organic additives has been predominantly empirical, and their mechanism of action remains incompletely understood. The precise nature of the additives and their concentrations are often confidential information held by the electroplating company. Additives are depleted during the plating process and must be regularly replenished. Electrolysis induces some decomposition of the organics, resulting in their occlusion in the deposit.^90^

Oxidation of tin(ii) is a problem in MSA systems, as well as in other electrolytes. Indeed, loss of tin(ii), due to its oxidation to tin(iv), will decrease deposition rates and change the composition of the tin–lead alloy. The oxidation of tin(ii) to tin(iv) causes an insoluble sludge to form in the electrolyte due to hydrolysis of tin(iv). This stannic sludge, mainly containing SnO_2_, is difficult to remove and waste-treat. Sludges can also result in rough deposits. The tin(iv) compounds also react with tin(ii), building up colloids, which can coagulate and form a precipitate in most cases.^91^ The dissolved oxygen in the solution seems to be the dominant factor leading to the oxidation of tin(ii) of the electrolyte, with formation of SnO_2_ particles.^92^ Anti-oxidants are usually included in tin and tin alloy based baths to inhibit the formation of tin(iv).^93^ Examples of anti-oxidants include hydroquinone, hydrazine, ascorbic acid, and 2-hydroxy-6-methylbenzenesulfonic acid (cresol sulfonic acid). One of the advantages of MSA for tin electroplating is that of all the common acid electrolytes, MSA-based aqueous solutions of tin(ii) have displayed the highest stability known toward oxidation to tin(iv).^5,94^

Tin and tin–lead alloys are solderable and, therefore, are used extensively in the electronics industry to bond electronic components. Tin and lead can be readily co-deposited because of their similar standard reduction potentials. Tin and tin–lead deposits must exhibit all of the following characteristics: good solderability and reflowability; low porosity; good corrosion resistance; and uniformity of alloy composition, thickness, smoothness, and appearance, over a wide current density range.^90^ In electronic applications, bright tin plating is not generally preferred since commercial processes produce high internal stress in the deposits along with high organic inclusions, which affect the solderability.^38^ Hence, smooth matte deposits with sound solderability and low resistivity are preferred.

MSA-based electrolytes are also applied in the electroplating of nickel, cobalt and their alloys. These metals are typically plated from a so-called Watts-type plating bath, consisting of a metal salt (in this case a methanesulfonate salt) and a high concentration of boric acid (H_3_BO_3_). The most commonly reported function of boric acid is as a buffer, preventing wide pH changes at the cathode–solution interface that could cause precipitation of metal hydroxides.^95^ Boric acid adsorbs to the electrode surface under certain conditions. This adsorption can decrease the active surface area and inhibit reduction reactions, as well as change the texture of the deposited film. Hence, boric acid also acts as a grain refiner impacting brightness, hardness, stress and adhesion. Most of the MSA-based electroplating baths for nickel and cobalt contain, in addition to the metal methanesulfonates and boric acid, also some sodium chloride.^96–98^ The role of chloride is twofold: it assists anode dissolution and it increases the diffusion coefficient of nickel ions, thus permitting a higher limiting current density.^99^

A mistake that a novice in the field of electrochemistry of aqueous MSA electrolytes can easily make is to use tap water or well water instead of deionized water for preparing the electrolyte solution or for compensation of evaporation losses of water in the electrolyte. Depending on the source, tap or well water can contain higher or lower concentrations of impurity elements. However, in all cases it will contain some amounts of chloride and sulfate anions. Particularly in the case of lead and tin–lead solder electroplating, adding tap or well water to the electrolyte will result in a steady increase of the chloride and sulfate concentrations to such an extent that at some moment the solubility limits of PbCl_2_ and PbSO_4_ will be exceeded, with precipitate formation as a result.

## Extractive metallurgy

The application of MSA in extractive metallurgy has been the topic of an earlier review paper by the present authors.^6^ Only the most important findings will be repeated here; the reader is referred to the cited review for more details. The justification for application of MSA for the recovery of metals from ores, concentrates, industrial process residues and urban waste is often the high solubility of the metal methanesulfonate salts compared to other metals and the fact that methanesulfonate salts are very suitable for electrolytes for electrowinning or electrorefining. The advantage of higher solubilities is not outspoken for all metals, as mentioned above in the section on the solubilities, but the major benefits are found in the processing of lead, tin and silver. Another advantage is the high solubility of calcium methanesulfonate compared to calcium sulfate. By reaction between calcium methanesulfonate and sulfuric acid in the raffinate after removal of the valuable metals, MSA can be regenerated and a pure gypsum precipitate can be recovered (*vide infra*).

### Copper hydrometallurgy

**Fig. 11:**
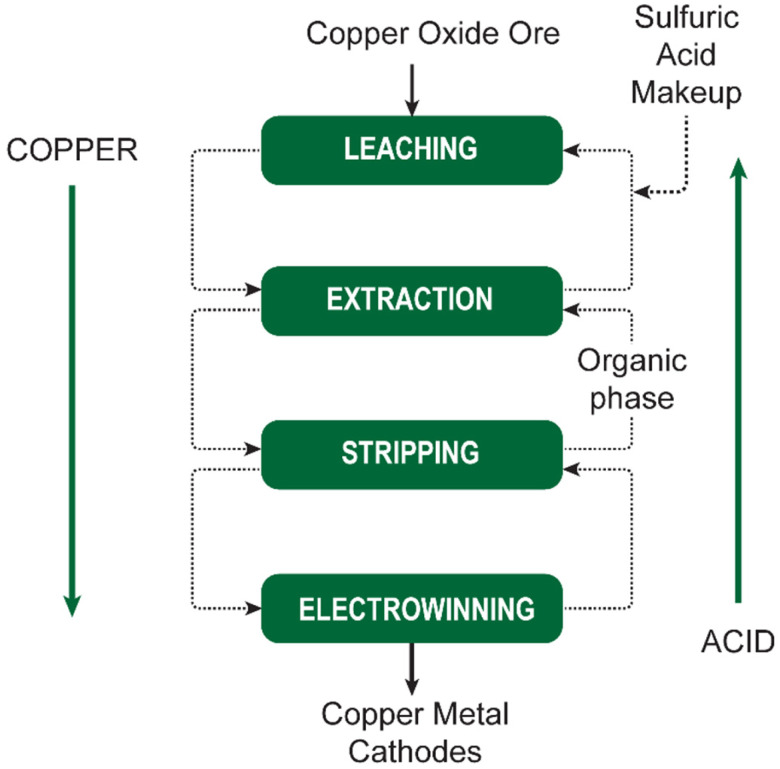
Schematic flowsheet of the leach–solvent extraction–electrowinning (L-SX-EW) process for recovery of copper from oxidic ores. Reproduced from ref. 102.

The *solvent-extraction*–*electrowinning* (SX-EW) process, sometimes referred to as the *leach solvent*–*extraction electrowinning* (L-SW-EW) process, represents the industrial benchmark for the extraction of copper from oxidic ores, because of its simplicity, elegance and limited environmental footprint.^100,101^ The process flowsheet is an almost completely closed cycle and, in theory, only copper oxide and electrical power are required to produce ultrapure copper metal. The flowsheet is composed of three interconnected closed circuits: the leaching, solvent extraction, and electrowinning circuits (Fig. 11).^102^ Oxidic copper ores are leached with a sulfuric acid solution, followed by selective removal of copper from the leach solution by solvent extraction with a copper-selective extractant. Copper ions are transferred to the organic phase with exchange of protons. This proton exchange regenerates the sulfuric acid in the aqueous solution so that is can be used to leach more copper ore. The copper is stripped from the loaded organic phase by the spent electrolyte of the electrowinning process. This stripping reprotonates the acidic extractant so that this can be reused in a new solvent extraction cycle. The copper is recovered from the stripped copper solution (called the *advance electrolyte*) by electrowinning, resulting in highly pure copper cathodes. Water is oxidized to oxygen gas at the anode and this oxidation reaction also generates protons. Overall, the acid that is consumed for copper leaching is regenerated during the electrowinning process.

In principle, MSA could replace sulfuric acid in the L-SX-EW process; the reaction mechanisms will remain the same, with the exception that methanesulfonate anions would substitute for (hydrogen)sulfate anions Because MSA is much more expensive than sulfuric acid, it is unlikely that MSA will be used for leaching of low-grade copper ores. Moreover, the copper in such ores is often solubilized by bacterial leaching (bioleaching) so that there is no need to add extra acid to the heap leaching process. On the other hand, for high-grade oxidic copper ores or oxidic copper concentrates that are typically leached by agitated leaching rather than by dump or heap leaching MSA could be considered as the lixiviant. Although limited information is available about the leaching behavior of such ores in MSA, it was reported that malachite, Cu_2_CO_3_(OH)_2_, can be readily dissolved in MSA.^103^ However, the gain in solubility of copper when MSA is used instead of sulfuric acid for leaching and supporting electrolyte in the electrowinning is rather limited; the aqueous saturation solubility (at 22 °C) of copper(ii) in the form of methanesulfonate is 2.00 M, whereas that in the form of sulfate is 1.35 M. It can be expected that the solubility of copper(ii) in both cases will sharply decrease with increasing acid concentration, although no data are available for copper(ii) at high MSA concentrations yet. However, investigation of copper electrorefining in MSA electrolyte is recommended to find out whether compact, high-purity copper cathodes could be obtained with less addition of additives than in sulfuric acid electrolytes.

The leaching of chalcopyrite (CuFeS_2_) is much more challenging than that of oxidic copper minerals. Although chalcopyrite is the most abundant copper mineral, it is very refractory towards acids under ambient conditions because of the formation of a surface passivation layer.^104^ MSA itself is a poor lixiviant for chalcopyrite because of its non-oxidizing nature. However, a mixture of MSA and hydrogen peroxide (H_2_O_2_) can efficiently leach the copper from chalcopyrite, provided that hydrogen peroxide is occasionally added during the leaching process to keep the oxidation–reduction potential (ORP) sufficiently high.^105^ Using hydrogen peroxide as an oxidant, the copper-extraction yield was approximately 60% after 96 hours at 75 °C. This yield could be boosted to 99% by the periodic addition of hydrogen peroxide over a longer period of time using 30 g L^−1^ MSA. Leaching with ferric ions yielded no more than 20% copper recovery. The sulfide ions were oxidized to elemental sulfur. Further studies indicated that the chalcopyrite leaching was very dependent on the hydrogen peroxide concentration.^106^ The reaction mechanism is diffusion controlled through a protective sulfur layer at lower temperatures, and controlled by the surface chemical reaction at temperatures in excess of 55 °C. Other oxidizing agents have been added to MSA to enhance the leaching kinetics of chalcopyrite in MSA. These include FeCl_3_,^107,108^ K_2_Cr_2_O_7_,^109^ and NaNO_3_.^109^ Although such studies are of interest from a fundamental point of view, as they help to reveal the leaching mechanism, they are less interesting for industrial applications because of the added costs and the more complex mixed-anion leachates.

The electrorefining of copper from MSA electrolyte has been investigated and the results were compared with those of electrorefining in sulfuric acid electrolyte.^110^ In all experiments with MSA electrolyte, the current efficiency for copper electrodeposition exceeded 99%, but the cell voltage and hence the specific energy consumption were distinctly higher than for the sulfuric acid electrolyte. However, the advantage of MSA electrolyte is that smooth copper cathodes could be obtained without the need of adding additives such as glue, thiourea or chloride. Also the silver and sulfur contents were lower in the cathodes obtained from MSA electrolyte. These preliminary results are favorable and further research on the use of MSA for electrorefining of copper is duly justified.

### Zinc hydrometallurgy

The exploration of the MSA potential for the extractive metallurgy of zinc has been restricted so far to leaching studies on different zinc ores. Smithsonite (ZnCO_3_) readily dissolves in MSA.^111^ As expected, the leaching rate is enhanced with increasing MSA concentration, reaction temperature, stirring speed, and decreasing particle size. The dissolution process follows the kinetic law of the shrinking core model. Similar results were obtained for the leaching of hemimorphite (Zn_4_Si_2_O_7_(OH)_2_·H_2_O) in MSA.^112^ Sphalerite (ZnS) can be leached efficiently by Fe(CH_3_SO_3_)_3_.^113^ A solution of 0.8 M Fe(CH_3_SO_3_)_3_ could extract 99.3% of zinc from sphalerite particles (particle size: 106–150 μm) after leaching at 70 °C for 96 hours. Zinc extraction was slightly promoted by the presence of oxygen and was significantly boosted by the presence of lattice iron in the (Zn,Fe)S sphalerite solid solution. Leaching of high-Fe sphalerite (13.94 wt% Fe) was about three times faster than for low-Fe sphalerite (0.40 wt% Fe). Fe(CH_3_SO_3_)_3_ was found to be more efficient than Fe_3_(SO_4_)_2_ or FeCl_3_ for leaching of sphalerite.

**Fig. 12:**
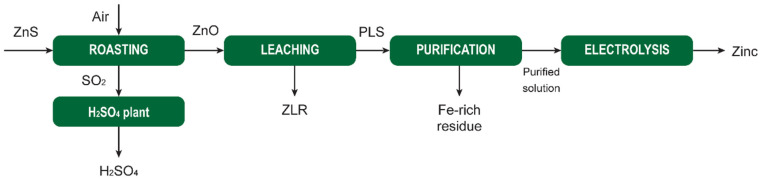
Simplified flowsheet of the Roast–Leach–Electrowinning (RLE) process in zinc hydrometallurgy. ZLR = zinc leach residue. Redrawn from ref. 115.

MSA presents a more promising prospect for the extraction of zinc as compared to copper. This is attributed to the fact that zinc sulfide concentrates invariably encompass substantial quantities of lead. The *Roast*–*Leach*–*Electrowinning* (RLE)^114^ process is the predominant method employed in the zinc industry. The RLE process accounts for over 85% of global zinc production. It leverages established technology within large-scale and energy-efficient facilities. The primary feedstock for these facilities is zinc sulfide concentrate, procured from mines worldwide. A majority of zinc producers utilize a blend of concentrates to ensure process stability and circumvent dependencies on a single mine. Within the RLE process, a zinc sulfide concentrate (ZnS) undergoes roasting to yield zinc oxide (ZnO), which is subsequently leached with sulfuric acid, and recovered from the purified ZnSO_4_ solution *via* electrowinning. The presence of iron ions in the leach solution impedes the zinc electrodeposition, necessitating a purification step prior to electrowinning. The form in which the iron is precipitated determines the nomenclature of the entire process: the jarosite, the goethite, or the hematite process. A simplified flow diagram of the RLE process is shown in Fig. 12. Independent of the type of iron-removal process, two residual streams are produced: (1) the iron-rich precipitate and (2) the *Zinc Leach Residue* (ZLR). Although the composition of the ZLR is highly dependent on the leaching steps and on the type of concentrate, it is mainly composed of lead (4–20 wt%), zinc (1.5–20 wt%), iron (6–23 wt%), calcium (2–4 wt%), and silver (300–1000 ppm). Therefore, this residue is a valuable secondary source of lead, zinc, and silver. The most common way of processing this residue is *via* pyrometallurgy, but such a process does not give direct access to the valuable silver. Therefore, there is interest in hydrometallurgical flowsheets for processing of ZLR.

**Fig. 13:**
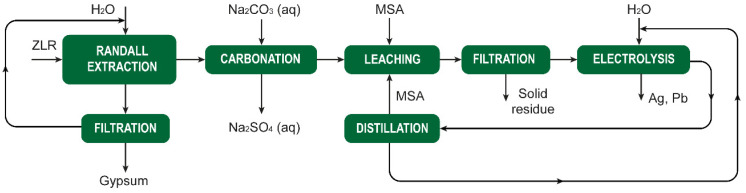
Conceptual flowsheet for the MSA-based recovery of lead and silver from zinc leach residue. Redrawn from ref. 115.

An MSA-based flowsheet was developed for the recovery of lead and silver from the leaching residue of the roast–leach–electrowinning process in the zinc industry (Fig. 13).^115^ This process takes advantage of the high solubility of lead(ii) and silver(i) methanesulfonate. The direct leaching of the ZLR with MSA resulted in a low lead-leaching efficiency and high co-dissolution of gypsum (CaSO_4_·2H_2_O). The gypsum was selectively recovered *via* Randall extraction by immersion in hot water, which was continuously reused. A desulfurization step with a Na_2_CO_3_ solution was utilized to convert PbSO_4_ into PbCO_3_, which can be easily dissolved in MSA. The carbonation was performed after the Randall extraction to avoid high Na_2_CO_3_ consumption by reaction with the gypsum still present in the residue. The leaching efficiency of lead drastically increased after the carbonation reaction. Nearly 80% of the lead and silver could be recovered. It was proposed to recover the lead and silver from solution by electrowinning, although this was not tested in practice. The authors suggested concentrating the MSA at the end of the process by evaporating part of the water, but they admitted that distillation is energy-intensive and that cheaper alternatives should be sought.

A leap in technological development would be an RLE process based on MSA rather than on sulfuric acid, because it could drastically reduce the volumes of waste generated by the zinc industry, as significantly less ZLR would be formed. Lead and calcium would be solubilized in the form of highly soluble methanesulfonate salts, rather than locked in the ZLR in the form of poorly soluble sulfates.

However, the development of such a new process cannot be achieved merely by substituting sulfuric acid with MSA in the flowsheet. The solution must undergo appropriate modifications given that the leachate will contain a significantly higher concentration of dissolved lead and other impurities. The jarosite process cannot be employed to eliminate iron from the flowsheet, as the absence of sulfate ions prevents the precipitation of basic double sulfates. It is highly probable that solvent extraction will assume a significant role in this novel MSA flowsheet for zinc hydrometallurgy.

### Lead hydrometallurgy

Sulfidic lead ores (galena, PbS) are typically processed by pyrometallurgy because the autogenous smelting of high-grade concentrates makes this process energy efficient. On the other hand, oxidic lead ores (PbO, Pb(OH)_2_, PbCO_3_, PbSO_4_) are not very suitable as feed for pyrometallurgical smelting operations because of the lack of intrinsic fuel value of their concentrates. Therefore, they should be processed *via* hydrometallurgy, by leaching, followed by electrowinning or precipitation. In the past, several hydrometallurgical processes for lead based fluoroboric and fluorosilicic acid have been developed, but these suffer from the issues that have been described in section on electroplating (*vide supra*). And similarly to the replacement of fluoroboric or fluorosilicic acid by MSA in electroplating applications, MSA-based hydrometallurgical processes for lead have been developed as alternatives for those based on fluorinated acids.

**Fig. 14:**
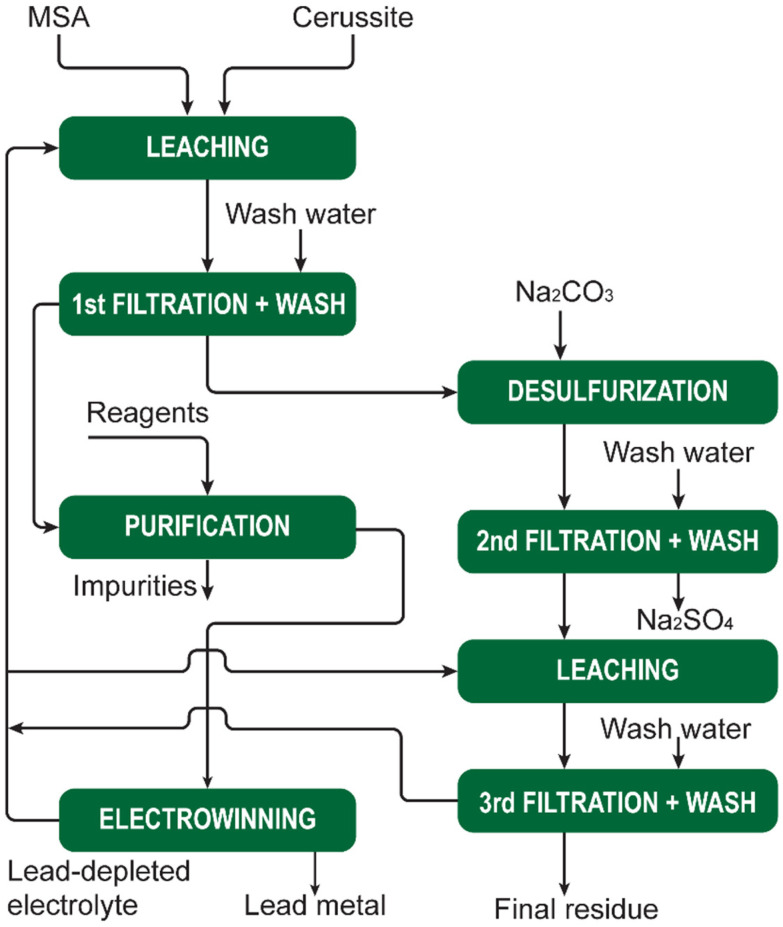
Schematic flowsheet for processing of cerussite concentrate by an MSA-based process. Redrawn from ref. 116 with permission from Elsevier. Copyright 2014.

The University of British Columbia (UBC, Canada), in collaboration with BASF (Germany), has developed an MSA-based process for treating oxidic lead ores.^116,117^ This process has been demonstrated for cerussite (PbCO_3_) concentrate. In the primary leach, cerussite was first leached with MSA and readily dissolved. The leaching kinetics were fast: most of the cerussite was dissolved in less than 5 minutes. The residue of the primary leach, which consisted largely of anglesite (PbSO_4_), was treated in a desulfurization step with Na_2_CO_3_ solution to convert the anglesite to cerussite. The desulfurized residue was leached with MSA in the secondary leach. In total, more than 98% of the lead present in the cerussite concentrate could be solubilized. The lead was recovered from the leach solution by electrowinning.^118^ The electrolyte had a concentration of 100–150 g L^−1^ of lead, as Pb(CH_3_SO_3_)_2_, and 0.25–0.5 M free MSA. A graphite anode and a stainless-steel cathode were used in the undivided electrolysis cell. A schematic representation of the flowsheet is shown in Fig. 14.

The process had to be adapted for the leaching of sulfidic lead ores (galena concentrates) because the direct leaching of PbS in MSA is inefficient and triggers the formation of toxic H_2_S gas.^119,120^ Galena can be oxidatively leached by Fe(CH_3_SO_3_)_3_ in MSA. By controlling the experimental conditions, the sulfide ions can be converted either to elemental sulfur or oxidized further to sulfate ions. The sulfate ions will react with the lead(ii) ions in solution to form a PbSO_4_ precipitate, but the latter compound can be desulfurized with Na_2_CO_3_ to PbCO_3_. The proposed flowsheet is similar to that for the processing of cerussite concentrate, with the difference being that the electrowinning is performed in a divided electrolysis cell to regenerate the Fe(CH_3_SO_3_)_3_ leaching reagent at the anode.

The BASF-UBC process has been further studied at the bench, pilot and demonstration scales on a lead concentrate of the Paroo Station lead mine in Western Australia, owned by LeadFX.^121^ LeadFX secured the process rights from BASF and UBC. The process involves a three-step leaching of a lead concentrate in MSA (“Leaching–Desulfurization–Releaching”), followed by electrowinning of lead metal. Cerussite is directly dissolved in the spent MSA electrolyte of the electrowinning circuit (step 1), but minor lead minerals such as pyromorphite must be processed first by sulfuric acid to convert them into PbSO_4_, which can be treated subsequently by Na_2_CO_3_ by desulfurization and conversion to PbCO_3_ (step 2). Step 3 is a re-leach with MSA of the PbCO_3_ formed in the desulfurization step. Galena can be leached by adding Fe(CH_3_SO_3_)_3_ to the MSA solution. Although Fe(CH_3_SO_3_)_3_ can be partially regenerated in the electrowinning circuit, regeneration is aided by the addition of hydrogen peroxide to the spent electrolyte. The advance electrolyte of the electrowinning circuit contains more than 300 g L^−1^ of dissolved lead. Cathode lead with a purity of more than 99.995% is produced at current densities between 300 and 400 A m^−2^. The electrowon lead cathodes are melted in an induction furnace and cast to lead ingots.

Whereas electrowinning is used to recover metals from solution after these metals have been solubilized from a solid material by a leaching process, the aim of electrorefining is to purify impure metals. In electrorefining of lead, impure lead anodes are electrochemically dissolved and pure lead metal is electrodeposited at the cathode.^122^ Impurities of more noble elements such as silver, gold and tellurium do not dissolve in the electrolyte and are recovered in the anode slime.^123^ Several MSA-based electrorefining processes have been developed to replace the conventional electrolytes containing fluoroboric or fluorosilicic acid.^124–126^ The electrochemistry of the lead electrodeposition during electrowinning and electrorefining is obviously very similar to that of lead during electroplating.

**Fig. 15:**
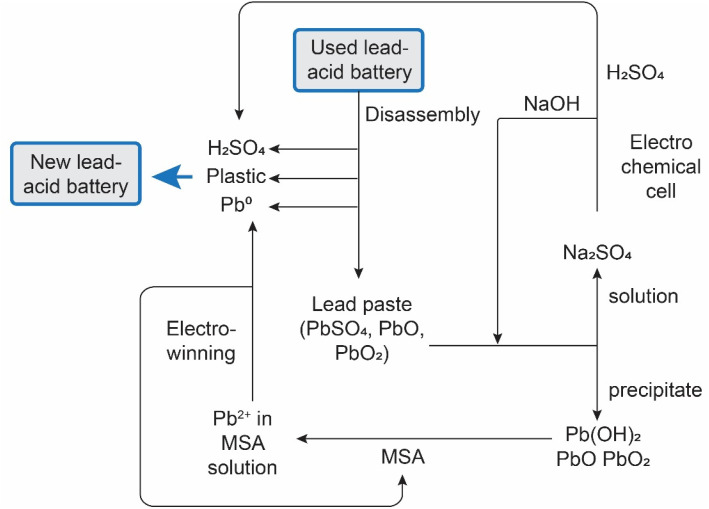
Conceptual flowsheet for the recycling of lead–acid batteries. Redrawn from ref. 128.

Also for lead–acid battery recycling, MSA offers a safer and environmentally friendly alternative to the well-established technologies based on fluoroboric or fluorosilicic acid. The challenge is to develop a method that can efficiently recover the lead from the lead paste in lead–acid batteries, which mainly consists of a mixture of PbSO_4_, PbO and PbO_2_.^127^ Metallic lead is easier to treat *via* smelting operations. Clarke *et al.* disclosed a process in which the lead paste is desulfurized by treating it with a NaOH solution, followed by leaching of the solid material with MSA.^128^ The lead can be recovered from the resulting lead(ii) methanesulfonate electrolyte by electrowinning (Fig. 15). Hydrogen peroxide can be added to MSA as a reducing agent to convert the insoluble PbO_2_ to soluble PbO.^129^ The PbSO_4_ desulfurization and PbO_2_ reduction steps can be combined by treating the lead paste by a solution containing a mixture of Na_2_CO_3_ and Na_2_SO_3_.^130^

It will become apparent to the reader that a number of the most promising applications of MSA are linked to those involving lead. We have already discussed the use of MSA as electrolytes for the electroplating of lead–tin solder and lead, as well as for the electrowinning of lead. Later in this review, we will discuss the use of lead(ii) methanesulfonate in redox flow batteries. This close association between MSA and lead can naturally be explained by the high solubility of Pb(CH_3_SO_3_)_2_, coupled with the advantageous properties of MSA systems. However, the fact that we provide extensive discussions on lead and its applications in green chemistry necessitates some justification, as not all researchers are convinced that lead has a future in clean technologies. Certainly, one must not downplay or disregard the risks associated with the unregulated release of lead into the environment. Lead is toxic and can have severe health impacts. However, lead can be safely managed in a controlled environment. The authors are persuaded that, given the necessary safety measures are implemented, lead should not be viewed as a problematic metal and that it plays a crucial role in clean technologies. What many policymakers and even scholars fail to realize is that lead is a pivotal facilitator of the circular economy.^131,132^ Lead metallurgy forms part of a complex industrial symbiosis system to manage intricate input materials (*e.g.*, Waste Electrical and Electronic Equipment or low-grade residues). Lead serves as the carrier phase in the recycling of numerous technology elements such as indium, tin, antimony, bismuth, tellurium, and also silver.^133,134^

Contrary to common belief, lead–acid batteries are not an outdated technology.^135^ Most electric vehicles still employ a lead–acid battery to supply 12 V power to all electric subsystems when the motor is not operational. Lead–acid batteries offer several advantages: they are reliable, robust, and considerably cheaper than lithium-ion batteries (LIBs). Lead–acid batteries are often more suitable for energy storage applications where cost is the primary concern. They are useful for stationary storage applications, both for standalone off-grid applications and grid-connected electricity storage systems.^136^ Lead-containing redox flow batteries could also be utilized for these applications (*vide infra*). Furthermore, spent lead–acid batteries are much simpler to recycle than LIBs. Lead–acid battery recycling is a mature technology, unlike LIB recycling, which continues to face a multitude of technical, economic, and environmental challenges.

### Silver hydrometallurgy

MSA solutions have been proposed as alternative for both conventional silver electrowinning and electrorefining from nitric acid solutions. The conventional Möbius electrolysis process for electrorefining of silver in nitric acid electrolytes suffers from problems that silver is cathodically deposited in the form of small dendrites with poor adherence on the cathode.^137^ In the Möbius process, they are periodically removed with scrapers and accumulate at the bottom of the electrolysis cell.^110^ To avoid contamination of the silver cathodes by anode slime particles, the anodes are placed in anode bags. At higher nitric acid concentrations in the electrolyte, the silver dendrites at the cathode will chemically redissolve and the nitrate ions are reduced at the cathode with release of toxic NO_*x*_ fumes. The cell design is complex because scrapers and anode bags are needed. The process is energy intensive because a cell potential of 2.3 to 7.0 V must be applied and the electrolyte bath is constantly heated to 40–55 °C. As a result, the energy consumption is 600 to 900 kW h per ton of silver metal produced.

By switching to an MSA electrolyte, a much more efficient silver electrorefining process was obtained (Table 4). It was found that an increase in silver and free acid concentration improved the quality of the cathode silver significantly. The electrolyte should contain in excess of 100 g L^−1^ of silver and at least 144 g L^−1^ of free MSA. The free acid concentration must be between 144 and 240 g L^−1^ for a minimum specific energy consumption; lower concentrations lead to higher energy consumption because of insufficient electrical conductivity, whereas very high free acid concentrations cause higher anodic polarizations or even anode passivation. Anode passivation becomes worse about 288 g L^−1^ (or 3 M) free acid. The mechanism of this anode passivation is not understood yet. Compact silver cathodes could be obtained in MSA, in contrast to the dendritic depositions in the nitric acid system. Neither scrapers nor anode bags were needed.

**Table 4:** Comparison of the performance of silver electrorefining by the Möbius process (HNO_3_) electrolyte and an MSA-based process^137^

Electrolysis data	Möbius process	MSA-based process
Cell voltage [V]	2.3–7	0.2–0.7
Current density [A m^−2^]	700–1000	150–450
Current efficiency [%]	>99	75–99.8
Specific energy consumption [kW h t_Ag_^−1^]	600–900	20–170
Silver content [g L^−1^]	100–200	100–150
pH control	Yes, necessary	No
Electrolyte temperature [°C]	40–55	Room temperature

As part of the development of a new MSA-based process for the refining of crude silver recycled from secondary resources, the dissolution characteristics of silver metal granules in MSA were studied.^138,139^ While MSA alone did not dissolve the granules, the addition of hydrogen peroxide as an oxidizing agent resulted in dissolution. Silver granules, containing approximately 94% of silver along small impurities of gold and PGMs, could effectively be leached by a mixture of MSA and H_2_O_2_. Leaching yields in excess of 90% were found, with solid-to-liquid ratios of up to 550 g L^−1^ and a threefold stoichiometric excess of H_2_O_2_. An optimum yield was obtained for temperatures between 65 and 85 °C. A significant selectivity for palladium was attained, with no more than 7% of the palladium undergoing co-dissolution. The primary constituents of the leaching residue were gold and unleached silver, accompanied by minor quantities of palladium and platinum. A correlation was noted between the solubility of silver(i) methanesulfonate and the concentration of free methanesulfonic acid (MSA) following the leaching process.^139^

### Urban mining

End-of-life consumer goods and especially waste of electrical and electronic equipment (WEEE) are valuable secondary resources for metals, both base metals and precious metals. The collection and recovery of metals from this waste fraction generated by human activities is called *urban mining*. Several studies have been devoted to the application of MSA in urban mining, addressing different types of waste such as photovoltaic cells, batteries, permanent magnets, fluorescent lamp phosphor waste… A detailed overview of these studies can be found in a previous review by the present authors,^6^ so that here only a short summary of the most important studies is given.

A pioneer in the application of MSA for recycling of metals is Dr Wolfram Palitzsch, who developed – at Loser Chemie (Germany) – an innovative process for recycling end-of-life thin-film photovoltaic (PV) modules, in which MSA plays an essential role.^140,141^ In contrast to conventional recycling technologies, the photovoltaic modules are not shredded so that the glass doesn't need to be broken. The sandwich structure of the modules is opened, and the glass panels are taken out in one piece. These glass panels are coated with a thin layer of a semiconductor material, such as cadmium telluride (CdTe), gallium arsenide (GaAs), copper indium selenide (CIS) or copper indium gallium selenide (CIGS). The molybdenum, indium tin oxide (ITO) and silver conducting layers and contacts can be dissolved in a mixture of MSA and hydrogen peroxide (50%). Silver can be recovered from the polymetallic solution by the precipitation of silver(i) chloride (AgCl) upon the addition of hydrochloric acid.^140,142^ The cleaned glass panels can be reused or used as a raw material in the production of floated glass. The dissolution step with MSA was also applied to the silver contacts of silicon solar cells.^143^ Palitzsch developed processes in which rare-earth elements (REEs) were leached by MSA from different types of REE-containing materials such as lamp phosphor waste, permanent magnets, lanthanum hexaboride (LaB_6_) cathodes, and the REEs were recovered from solution by precipitation of the REE oxalates by addition of oxalic acid to the leach solution.^144,145^ By this reaction, MSA was regenerated (*vide infra*).

In processes for recovery of MSA from urban waste, MSA is often combined with hydrogen peroxide (H_2_O_2_). Although MSA is a non-oxidizing acid, the combination of MSA and H_2_O_2_ provides a strongly oxidizing mixture, that can be used to dissolve less reactive metals such as silver. The dissolution of silver by a mixture of MSA and H_2_O_2_ is described previously. Mixtures of MSA and H_2_O_2_ have also been used to selectively dissolve the solder (lead–tin alloy) for the dismantling of electronic components from waste printed-circuit boards (PCBs).^146^ The MSA and H_2_O_2_ concentrations and the reaction time had to be optimized to selectively dissolve the lead and tin, with a minimum co-dissolution of the copper present underneath the solder. After the dissolution of the solder, the electronic components could be easily separated from the PCBs. The use of H_2_O_2_ significantly accelerated the dismantling process.^147^ Oxidative leaching with mixtures of MSA and H_2_O_2_ was also applied for recovery of LiFePO_4_ cathode material; oxidation of iron(ii) to iron(iii) converts the material in FePO_4_ with release of lithium ions to the solution.^148^

Furthermore, hydrogen peroxide can act as a reducing agent towards strongly oxidizing compounds so that the MSA + H_2_O_2_ combination can also be applied to reductive leaching. Reductive leaching is used for dissolution of cathode materials of lithium-ion batteries where cobalt(iv) and cobalt(iii) must be reduced for efficient leaching. The MSA + H_2_O_2_ combination was adopted for dissolution of LiCoO_2_ cathode materials.^149^ In the presence of a small amount of hydrogen peroxide as a reducing agent (0.9 vol%), lithium and cobalt could be leached very efficiently by a 1 M MSA solution. MSA performed much better than other organic acids that were tested (citric acid, malonic acid, succinic acid and oxalic acid). Furthermore, the method was applied to black mass from spent lithium-ion batteries with LiCoO_2_ cathode materials. MSA + H_2_O_2_ performed well for the recovery of cobalt, nickel and manganese from black mass prepared from shredding of spent lithium-ion batteries with NMC cathode materials.^150^ A comparison between the leaching efficiencies of MSA and sulfuric acid showed that the two acids yielded similar results, provided solutions of the same acid strengths are compared: 1 M MSA *versus* 0.5 M H_2_SO_4_. It is important to note that, when hydrogen peroxide is acting as a reducing agent, it is oxidized to oxygen gas. The evolution of oxygen gas can cause experimental difficulties when the reaction is carried out at on a larger scale in a reactor, because excessive foaming of the solution can occur.

Since MSA has a very low vapor pressure, pure MSA can be used as a lixiviant for leaching at high temperatures without the need of an autoclave. This type of MSA application has been illustrated for the recovery of terbium from refractory lamp phosphor waste. The lamp phosphor waste powder fraction of end-of-life fluorescent lamps is a complex mixture of inorganic compounds. The largest fraction is the white phosphor (Sr,Ca)_10_(PO_4_)_6_(Cl,F)_2_:Sb^3+^,Mn^2+^ (HALO). HALO does not contain REEs, but it is relatively rich in antimony. The other phosphors are the red phosphor Y_2_O_3_:Eu^3+^ (YOX), the green phosphors LaPO_4_:Ce^3+^,Tb^3+^ (LAP), CeMgAl_11_O_19_:Tb^3+^ (CAT), (Ce,Gd)MgB_5_O_10_:Tb^3+^ (CBT), and the blue phosphor BaMgAl_10_O_17_:Eu^2+^ (BAM). Additionally, the waste powder contains fine glass particles and alumina from the binder. Most research studies have focused on the recovery of REEs from YOX phosphor, containing high yttrium and europium concentrations. YOX and HALO are easy to dissolve in dilute acids, but the other phosphors are much more refractory. The residue after recovery of YOX is often landfilled, although it still contains large amounts of valuable terbium. LAP is the most interesting terbium-containing compound in the waste fraction, because of its high terbium content and the fact that it is easier to dissolve than CAT and CBT. Nevertheless, harsh reaction conditions are required to dissolve LAP, such as treatment with concentrated sulfuric acid at temperatures above 250 °C or alkali fusion. Hence, a milder MSA-based process was developed to dissolve the terbium present in LAP phosphor waste.^151^ The process was applied on a residue obtained after leaching lamp-phosphor waste with a H_2_SO_4_ solution. By leaching with anhydrous MSA at 180 °C, the LAP could be fully dissolved in about 1 hour. Lower temperatures in combination with prolonged reactions times also allowed complete dissolution of LAP. MSA did not dissolve the green phosphors CAT and CBT. Further processing of the pregnant leach solution by solvent extraction, and precipitation by oxalic acid, followed by calcination, made it possible to obtain the purified oxides Tb_4_O_7_, CeO_2_ and La_2_O_3_. This work was further extended to the development of a closed-loop, selective process for the recovery of the three main phosphors of the lamp-phosphor waste (HALO, YOX, and LAP) using only MSA as the lixiviant.^152^ In this regard, it is noteworthy that with the emergence of LED lamps, the use of fluorescent lamps has swiftly declined, suggesting a significant reduction in the market demand for closed-loop recycling schemes for lamp phosphors. This issue is prevalent in the field of urban mining, as the metals present in the currently collected waste fractions do not necessarily align with the metals needed in contemporary products being marketed today. These discrepancies between supply and demand pose a substantial obstacle to the realization of the circular economy model.

## Redox flow battery electrolytes

Since recently, MSA and methanesulfonate salts are finding applications in electrolytes for redox flow batteries (RFBs). The RFB is a type of rechargeable battery where chemical energy is stored in two soluble redox couples that are pumped through the electrochemical cell on separate sides of a membrane.^153^ An RFB fundamentally consists of three primary components. The first component is the cell, which is composed of a membrane and two electrodes. This is the site where redox reactions transpire. The second component is constituted by the electrolyte tanks, which serve to store the liquid electrolytes. The energy capacity of the RFB is a function of the volume of the electrolyte. The third component encompasses the pumps utilized to circulate the liquid electrolytes throughout the system. In contrast to conventional batteries, where energy is stored within the battery material itself, in RFBs, energy is stored in the electrolyte. The capacity of the battery (*i.e.*, the total amount of energy that can be stored) is determined by the size of the electrolyte storage tanks, implying that the system can be readily scaled up. The power of the RFB (*i.e.*, the rate at which electric energy is transferred) is determined by the size of the membrane. The capacity and power are decoupled, permitting independent sizing of capacity and power. Practical applications often necessitate high currents and voltages. To accomplish this, multiple cells can be stacked in electrical series to augment voltage, and the stacks can be electrically connected in parallel to produce high currents. RFBs are particularly well-suited for large-scale energy storage applications, including grid storage and the integration of renewable energy sources. However, they typically exhibit lower energy efficiency compared to other types of batteries, rendering them currently unsuitable for mobile applications. Hybrid flow batteries amalgamate a solution-based redox pair with a reaction involving the electrode surface and solution electrode (such as metal deposition/stripping or gas evolution/reduction).

The best known commercial RFB is the *all-vanadium flow battery* (VRB), that is based om the vanadium(ii)/vanadium(iii) and vanadium(v) redox couples for the negative and positive half-cell solutions, respectively.^154^ Originally, sulfuric acid was chosen as the supporting electrolyte.^155^ To minimize the volume of electrolyte needed for a particular energy-storage capacity, the vanadium concentration must be as high as possible. The solubility of each of the vanadium species over the expected operating temperature range of the VRB will determine the maximum vanadium concentration that can be used to avoid precipitation of vanadium with potential blockages in the stacks and pumps. Studies have shown that the solubilities of vanadium(ii), vanadium(iii) and vanadium(iv) decrease with increasing sulfuric acid concentration and with increasing temperature.

The reduced solubility at higher acid concentrations is a consequence of the common-ion effect because each of these species precipitate as vanadium sulfate salts. However, for vanadium(v) solutions, an opposite trend is observed: an increase in solubility with increasing sulfuric acid concentrations and decreasing temperature. This behavior is explained by the observation that vanadium(v) does not precipitate as a sulfate and, instead, undergoes thermal precipitation, according to the endothermic reaction:

23 2VO_2_^+^ + H_2_O ⇄ V_2_O_5_↓ + 2H^+^

The solubilities of the different vanadium species limit the maximum vanadium concentration in the electrolyte to 1.6–1.8 M for an operating temperature between 10 and 40 °C. In order to reach higher vanadium concentrations, different inorganic and organic precipitation inhibitors have been tested to stabilize supersaturated solutions of high vanadium concentration during the charge–discharge cycles of the VRB. In order to increase the vanadium concentration and to extend the operational temperature of the electrolyte, supporting electrolytes other than sulfuric acid have been tested.

Unsurprisingly, MSA-based supporting electrolytes have garnered interest from researchers dedicated to advancing RFB technology, despite the relatively limited number of studies conducted thus far. The first of these studies focused on vanadium salts in mixed MSA/H_2_SO_4_ supporting electrolytes, where a vanadium(iv) concentration of 2 M could be reached in a mixture of 1.5 M MSA and 1.5 M H_2_SO_4_.^156^ The addition of MSA also improved the redox reaction kinetics and the mass transport rate of the vanadium(iv)/vanadium(v) redox couple. With increasing MSA concentration, an improved solubility and stability of the vanadium ions were observed in similar electrolytes. However, this increase also negatively affected the solution's resistance and electrochemical kinetics. MSA acts as a precipitation inhibitor for vanadium in sulfuric acid supporting electrolytes.^157^ An electrolyte of 2 M vanadium(iv) in a 7 M MSA solution showed excellent reversibility and a slightly higher energy efficiency than an electrolyte comprising 2 M vanadium in 4 M H_2_SO_4_.^158^ In a study on MSA supporting electrolytes for zinc-vanadium RFBs, vanadium(iv) concentration as high as 3 M were studied in 2.0, 4.0 and 6.0 M MSA solutions.^159^ The vanadium concentration could be enhanced to even 4 M, but then the viscosity of the solution became too high. The solutions were found to be stable for months in the temperature range between 20 and 40 °C.

**Fig. 16:**
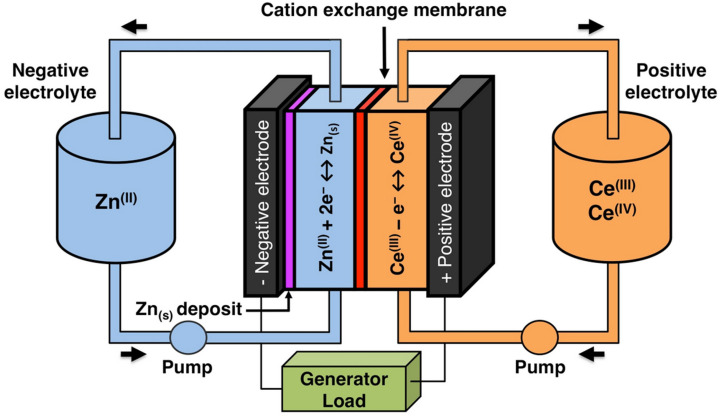
Diagram of a divided zinc–cerium redox flow battery. Source: wikipedia.^168^

Inspired by the work of Kreh *et al.* on the use of cerium(iv) methanesulfonate solutions as electrogenerated oxidizing agent in organic electrosynthesis,^53,160^ cerium(iii)/cerium(iv) in MSA has been considered as an electrolyte for the positive side in high-energy density RFBs.^161,162^ The most extensively studied RFB comprising cerium in MSA is the zinc-cerium hybrid RFB.^163–165^ In their discharged states, zinc-cerium RFBs are typically operated with 1.5 M zinc(ii) methanesulfonate in 1 mol dm^−3^ MSA on the negative side and 0.8 M cerous methanesulfonate in 4 M MSA on the positive side.^166^ The cell has the highest open-circuit cell potentials amongst aqueous RFBs, which can exceed +2.4 V at full charge. In the development of cerium-based RFBs one has to find a balance between electrode transfer kinetics and solubility of the cerium salt: the electrode transfer kinetics are improved at higher MSA concentrations, but higher MSA concentrations also lead to lower solubilities of cerium(iii) methanesulfonate and hence to a lower energy density of the electrolyte. Although a lot of research has been devoted to the development of zinc-cerium RFBs, this type of RFB has not been a commercial success yet. It was developed by the company Plurion Inc., but this RFB type never reached the market. A 2 kW system was installed in Plurion's testing facility in Scotland in 2007.^167^ In their divided zinc-cerium RFB, Nafion® cation exchange membranes were used to separate the two half-cell compartments (Fig. 16). The electrodes were made of glassy carbon. The system made use of MSA as the supporting electrolyte to yield reasonable solubilities for both the zinc (>2 M) and cerium species (about 0.8 M) in both oxidized and reduced forms. The RFB operated at 60 °C and under constant voltage discharge. The discharge current densities were up to 100 mA cm^−2^ with coulombic efficiencies of more than 70% over 60 cycles. In 2008, Plurion Inc. suffered a liquidity crisis and the company was formally dissolved in 2012.^168^ There are several issues that hamper the development of a successful zinc-cerium RFB.^166,169^ On the negative cell side, the energy efficiency of zinc electrodeposition is decreased by competition with the hydrogen evolution reaction. Several additives have been tested to suppress this hydrogen evolution reaction.^167,170^ There is also the issue of corrosion of the electrodeposited zinc metal in the MSA electrolyte. As regards the positive cell side, the limited chemical stability of the electrode material in the presence of the strongly cerium(iv) is a matter of concern. Another issue is the parasitic oxygen evolution reaction at the positive electrode. The elevated operational temperatures make it difficult to operate this RFB at remote locations. Likewise, this RFB type is suboptimal for grid-scale applications.^171^ The use of elevated temperatures is required to boost the sluggish kinetics of the cerium(iv)/cerium(iii) redox couple.^172^ On the other hand, from the point of view of the critically of the materials, the zinc-cerium RFB is advantageous. Zinc and cerium are both earth-abundant elements. The wide available of the rare-earth element cerium might be a surprise, but the abundance of cerium in the earth crust is comparable to that of copper, and large volumes of cerium are coproduced with the mining and refining of neodymium for rare-earth permanent magnets. In fact, there is an oversupply of cerium and finding new large volume applications for cerium is essential for mitigating the *Balance Problem* in rare-earth markets.^173,174^

Another type of RFB with MSA supporting electrolytes is the vanadium-cerium RFB, which is, in contrast to the zinc-cerium battery, not a hybrid flow battery, but a genuine RFB with all the involved redox-active species in dissolved form. In the initial designs of vanadium-cerium batteries, electrolyte contamination was a significant issue due to cation cross-over. This was primarily because a cation-exchange membrane was employed as a separator, which allowed vanadium ions to migrate into the cerium electrolyte and *vice versa*.^175,176^ This contamination leads to fast capacity fading. Sankarasubramanian *et al.* developed an MSA-based vanadium-cerium battery in which an anion-exchange membrane, made of a polystyrene-*block*-poly(ethylene-*ran*-butylene)-*block*-polystyrene (SEBS) triblock copolymer, was used as a separator to avoid cation cross-over.^177^ The capacity fading was only 2.4% over 100 cycles. A hydrogen-cerium RFB with an MSA-based electrolyte was developed to take advantage of fast, reversible kinetics and the fact that hydrogen is an abundant element.^178^ The RFB had a surprisingly low self-discharge.

Given the high solubility of lead(ii) methanesulfonate compared to other lead salts and the fact that the lead(0)/lead(ii) redox couple has been used in hybrid flow batteries, it is not surprising that MSA-based RFBs involving the lead(0)/lead(ii) redox couple have also been investigated. Lead–iron RFBs are promoted as making use of cheap and abundant elements. For instance, Zeng *et al.* developed an RFB using 0.6 M Fe(CH_3_SO_3_)_2_ in 2.0 M MSA as the positive electrolyte and 0.3 M Pb(CH_3_SO_3_)_2_ in 2.0 M MSA as the negative electrolyte.^179^ Although the RFB showed fast electrochemical kinetics and a stable cycle performance, the open-circuit cell potential was only 0.93 V. An undivided flow battery based on the Pb(ii)/Pb(0) and PbO_2_/Pb(ii) redox couples in aqueous MSA solutions has been tested.^180,181^ This is a hybrid flow battery in which solid lead metal is electroplated onto the negative electrode and solid PbO_2_ is electroplated onto the positive electrode during charging. During discharge, the solid phases are electrochemically dissolved back into the electrolyte as Pb(ii), the active species in both half-reactions. Hence, in principle, this RFB can be operated in an undivided manner. However, it was found that a divided call works much better, because the separator acts as a physical barrier to prevent failure by electrical shorting of uneven deposit growths. Furthermore, when an ion-exchange membrane is used, electrode-specific additives may be included in the two electrolytes.^182,183^ This type of battery is called the *soluble lead flow battery* (SLFB), because, unlike for the conventional lead–acid battery that is based on sulfuric acid, lead is highly soluble in the MSA electrolyte of the SLFB. Notwithstanding its low energy density of 20–40 W h L^−1^, the SLFB is a promising type of RFB, as it has a simple architecture making it relatively robust, and a lifetime of 2000 cycles demonstrated at the cell level. Also, the electrolyte is manufacturable directly from spent lead–acid batteries.^184^ A possible application of SLFBs could be in photovoltaic microgrids. In a cost analysis, MSA was predicted to be the largest cost component of the SLFB, with the graphitic bipolar plates the second largest contributor to the costs, so that the availability of cheaper MSA could make the SLFB economically more feasible.

## Recycling and regeneration of MSA

Electrochemical processes have been developed for the regeneration of MSA from spent electrolyte baths containing methanesulfonate salts.^5,185^ The MSA is generated by electrowinning of the metal from the metal methanesulfonate solution, either in a divided or an undivided electrochemical cell. The acid is generated by oxidation of water at an inert anode and/or by hydrolytic reactions of an anodically generated higher oxidation state of the metal cation. The anode is typically an IrO_2_ coated titanium inert anode and the anode reaction is:

24 2H_2_O → O_2_ + 4H^+^ + 4e^−^

The cathode reaction is the electrodeposition of metal:

25 M^*n*+^ + *n*e^−^ → M

**Fig. 17:**
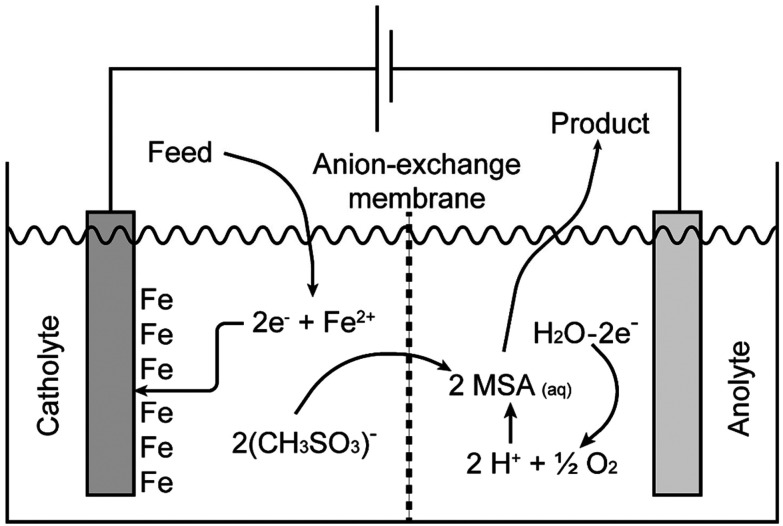
Divided electrochemical cell for recovery of MSA from a Fe(CH_3_SO_3_)_2_ solution. The cell is divided by an anion-exchange membrane that allows transfer of the MSA anion from the cathode to the anode compartment, where is combines with the protons formed by oxidation of water. Redrawn from ref. 5 with permission from the Royal Society of Chemistry, copyright 1999.

An undivided electrochemical cell works well only with metal ions that can be easily deposited from acidic solutions such as copper(ii). It is not recommended for other metal ions, such as iron(ii), cobalt(ii) and nickel(ii). The preferred setup for obtaining pure MSA is a divided electrochemical cell with an anion exchange membrane. The metal methanesulfonate solution is charged to the cathode compartment of the divided electrochemical cell and a diluted MSA solution to the anode compartment. The electrochemical process is optimized for electrodeposition of metal on the cathode and for oxygen formation on the anode. The methanesulfonate anions, which are freed up by the electrodeposition of the metal, pass through the anion-exchange membrane (anion-selective membrane) into the anode compartment. As such, they close the electric circuit of the cell. Upon completion of the electrochemical process, a pure MSA solution is contained in the anolyte. A schematic representation of the divided electrochemical cell and the occurring chemical reactions are shown in Fig. 17. Anion-exchange membranes are thin sheets of polymeric material wherein the polymer structure contains fixed ammonium and/or amine groups distributed throughout the backbone. Anion-exchanging ammonium groups are distributed in porous channels dispersed throughout the membranes structure. These porous channels are of molecular dimensions, and they are responsible for the selective transfer of anions. If the metal methanesulfonate solution in the cathode compartment is too acidic for efficient electrodeposition of the metal, the cathodic electrode process can be adjusted in such a way that hydrogen formation becomes dominant, and this reaction consumes protons from the cathode compartment. The net result of the divided cell electrowinning process during this early stage is simply transfer of acid from the cathode compartment to the anode compartment. After sufficient acid has been transferred from the catholyte into the anolyte, metal deposition may begin to occur.

The electrochemical process can be run in continuous mode. Here, the catholyte stream is continuously recycled while being maintained at constant metal salt concentration by additions of solid salt and/or concentrate. The anolyte stream contains the generated MSA. By circulating the anolyte, the MSA concentration is steadily increasing. Cathodic metal deposits are regularly removed. MSA regenerated by such a process from washing waters of an electroplating process is of a quality that is high enough to be reused for preparing fresh electrolyte for an electroplating bath.^186^ MSA concentrations up to 150 g L^−1^ (1.56 M) could be achieved.

In hydrometallurgical MSA flowsheets where NaOH is used as a base to control the pH, the final raffinate contains significant concentrations of Na(CH_3_SO_3_), just like sulfuric acid flowsheets lead to formation of Na_2_SO_4_ solutions. Major research efforts have been made to develop processes for salt splitting. The salt splitting of Na_2_SO_4_ into H_2_SO_4_ and NaOH is an attractive approach because it solves the issue of Na_2_SO_4_ waste, while simultaneously regenerating the acid and base consumed in the process. Different electrochemical membrane processes have been developed to salt split Na_2_SO_4_. These are often based on electrodialysis, such as *Bipolar Membrane Electrodialysis* (BMED).^187–189^ To the best of our knowledge, no studies on the salt splitting of sodium methanesulfonate have been performed yet.

The high solubility of methanesulfonate salts compared to sulfate and chloride salts can be exploited for regeneration of MSA from solutions of its salts. For instance, addition of sulfuric acid to an aqueous solution of calcium(ii) methanesulfonate will precipitate CaSO_4_·2H_2_O (gypsum), while MSA is being regenerated:

26 Ca(CH_3_SO_3_)_2_ + H_2_SO_4_ + 2H_2_O → CaSO_4_·2H_2_O↓ + 2CH_3_SO_3_H

Silver can be recovered from MSA solutions by addition of hydrochloric acid; poorly soluble silver(i) chloride will be precipitated, with regeneration of MSA:^190^

27 Ag(CH_3_SO_3_) + HCl → AgCl↓ + CH_3_SO_3_H

In a similar way, lead can be recovered:^5^

28 Pb(CH_3_SO_3_)_2_ + 2HCl → PbCl_2_↓ + 2CH_3_SO_3_H

Rare-earth elements (REEs) can be recovered from MSA solutions by addition of oxalic acid:^144,145^

29 2REE(CH_3_SO_3_)_3_ + 3H_2_C_2_O_4_ → REE_2_(C_2_O_4_)_3_↓ + 6CH_3_SO_3_H

After solid–liquid separation, an aqueous solution of MSA is obtained and the concentration of MSA can be increased by evaporation of part of the water, for instance by distillation with heat recovery.

## Comparison with other acids

Throughout this review, the characteristics of methanesulfonic acid (MSA) have been contrasted with those of other strong acids, including sulfuric, hydrochloric, and nitric acid. This section summarizes this information and juxtaposes the properties of MSA with other acids. MSA exhibits high thermal stability, low vapor pressure, and is odorless: these are traits it shares with concentrated sulfuric acid. However, unlike concentrated sulfuric acid, which is an oxidizing, strongly dehydrating, and sulfonating agent, MSA does not possess these properties (although pure MSA is hygroscopic). Hydrochloric and nitric acids are relatively volatile and emit a strong odor. Nitric acid is an oxidizing acid, while hydrochloric acid is highly corrosive to metals used in construction materials. MSA does not exhibit these characteristics. Owing to its redox stability, MSA is highly suitable as a supporting electrolyte for electroplating, electrowinning, or electrorefining of metals. This is less applicable for hydrochloric acid, due to the formation of chlorine gas at the anode, and nitric acid, due to the formation of NO_*x*_ gases at the cathode.

The main differences between MSA and phosphoric acid (H_3_PO_4_), is that phosphoric acid is a weak, triprotic acid, and that most of the phosphate salts are poorly soluble in water.

Fluoroboric acid (HBF_4_) and fluorosilicic acid (H_2_SiF_6_) share with MSA the high solubility of their lead(ii) and tin(ii) salts, so that fluoroboric and fluorosilicic acid can also be used as electrolytes in the electroplating, electrowinning and electrorefining of lead, tin and lead–tin solder. The main issues with these fluorinated acids is that they are not stable in water and undergo hydrolysis with release of hydrofluoric acid. Whereas waste water treatment of spent MSA electrolytes is relatively simple and safe, this is not the case for electrolytes based on fluoroboric and fluorosilicic acid.

Carboxylic acids such as formic or acetic acid are much weaker acids than MSA. Whereas MSA is non-volatile and odorless, these carboxylic acids are quite volatile and have a strong smell. Formic acid is a reducing agent and can be readily oxidized to carbon dioxide, whereas acetic acid is poorly resistant to oxidizing and reducing conditions. On the other hand, these acids share with MSA two key properties: biodegradability and high solubility of metal salts. For instance, lead(ii) acetate is highly soluble in water. However, metal acetates are not very suitable for electrochemical applications because of the redox instability of the acetate anion.

There is considerable research interest in utilizing citric acid in hydrometallurgical applications, where MSA could also be employed. Citric acid, a weak organic acid, can form robust complexes with metal ions in solution due to its chelating effect. However, unlike MSA, citric acid is a solid at room temperature and lacks stability at high temperatures and in the presence of oxidizing agents. Despite its chelating effect, citrate metal salts are often poorly soluble. For example, the solubility of calcium citrate is merely 8.5 g per 1000 mL of water at 18 °C. Consequently, citric acid can eliminate minor amounts of scale but is incapable of maintaining large quantities of calcium in solution. A widely recognized claim in favor of citric acid is its production from renewable resources *via* fermentation, suggesting that citric acid is a sustainable and renewable chemical.^191,192^ However, virtually all citric acid available in the market is of food-grade purity. The traditional method for purifying citric acid involves its recovery from the fermentation broth by precipitation with Ca(OH)_2_, followed by the release of citric acid from the precipitated calcium citrate through the addition of sulfuric acid.^193,194^ Hence, the purification of citric acid consumes large amounts of chemicals and generates significant volumes of gypsum waste.

In many applications, especially as an acid catalyst in organic synthesis, *p*-toluenesulfonic acid (PTSA) behaves very similarly to MSA. However, as explained above, MSA has the advantage over PTSA to be a liquid at room temperature and, hence, is more convenient to handle in chemical unit operations. Moreover, the presence of a phenyl group in PTSA makes it somewhat less environmentally friendly than MSA. The PTSA has a higher molecular mass than MSA so that for similar masses of salt, the metal content in an PTSA salt is lower than in the corresponding MSA salt.

At first sight trifluoromethanesulfonic acid (triflic acid, CF_3_SO_3_H) looks very similar to MSA since there is only substitution of a trifluoromethyl group for the methyl group. Still, the properties of triflic acid and MSA are very different. Triflic acid (p*K*_a_ = −5.5) is way more acidic than MSA (p*K*_a_ = −1.9), and it can be labeled as a ‘*super acid*’, *i.e.*, an acid that has a higher acidity than concentrated sulfuric acid. Since triflic acid is such a strong acid, its conjugated base, the triflate or trifluoromethanesulfonate anion, is a very weak base and even much more weakly coordinating than the methanesulfonate anion. Trifluoromethanesulfonate salts (triflates) are stronger Lewis acids than the methanesulfonate salts (mesylates), hence their use as Lewis acid catalysts in organic synthesis.^195^ Triflic acid is a much more aggressive acid than MSA and, as a result, more safety precautions need to be taken. There are also environmental concerns due to the fluorine content of triflic acid, since fluorinated compounds are poorly biodegradable. Triflic acid finds some applications in niche applications in hydrometallurgy such as the recycling of thoria (ThO_2_) from thorium-based nuclear-fuel production scrap.^196,197^

## Critical reflection on the green character of MSA

MSA is endorsed by numerous scholars and industry associates as a green acid that harbors immense potential for the advancement of novel clean technologies. These range from its use as catalysts in organic synthesis, to electrolytes for metal electroplating, to a lixiviant for metal recycling *via* urban mining, and even its application in redox flow batteries. This Tutorial Review delineates many of the merits of MSA over strong mineral acids and organic acids. Nonetheless, the authors are fully aware of the necessity for caution to avoid overstating the benign properties of MSA, lest we repeat the errors committed by researchers in the realm of *ionic liquids* (ILs) and *deep-eutectic solvents* (DESs). ILs and DESs have been extolled in such emphatic terms that a novice in the field could be easily misguided, forming the impression that these solvents possess such extraordinary properties that they can address all challenges in chemistry and beyond. It is only of late that some dissenting opinions have emerged from within this research domain.^198–200^ Consequently, it is imperative to critically evaluate the benefits of MSA and to acknowledge that MSA may also have certain limitations.^201^

Evaluating the sustainability of MSA necessitates a comprehensive consideration of the energy efficiency, resource utilization, and environmental footprint of the MSA production process. This evaluation should extend beyond merely accounting for greenhouse gas emissions. The old industrial processes for MSA production, which relied on the oxidation of mercaptans (thiols), were not environmentally friendly and even posed explosion risks.^5^ The chlorine-free MSA production process developed by BASF marked a positive advancement, yet it is the direct sulfonation of methane that truly offers the potential for a sustainable process.^9^ However, even for this reaction, the sustainability metrics will be significantly more favorable for methane derived from biogas as compared to methane sourced from fossil natural gas.

The biodegradability of MSA is often highlighted as one of its primary advantages. Incidental leaks of MSA may not be viewed as an environmental threat, as all MSA molecules eventually break down into CO_2_ gas and sulfate ions. It is evident that this biodegradation could contribute to greenhouse gas emissions, but fewer individuals are aware that the release of sulfate ions could also pose challenges. Numerous countries rightfully enforce stringent restrictions on the levels of sulfate in liquid effluents discharged into the environment.^202^ Sodium sulfate (Na_2_SO_4_) in waste water is a growing concern, not just for the chemical industry, but also for the mining and metallurgical sectors. Given that substantial quantities of sodium sulfate are generated during the production of cathode active materials for lithium-ion batteries, sodium sulfate is also a concern for the battery industry. While various solutions to the sodium sulfate problem have been suggested, such as salt splitting into sodium hydroxide and sulfuric acid by bipolar membrane electrodialysis,^203,204^ there is still no universally accepted solution to this issue.^205^ Even though the treatment of MSA effluent is simpler than that of fluoroboric and fluorosilicic acid, it is crucial not to overlook that MSA spills into the environment will ultimately result in elevated sulfate concentrations in surface waters, which must be prevented.

The exceptional solubility of metal methanesulfonates, a key advantage of MSA chemistry, has been repeatedly emphasized in this review. However, this high solubility is only applicable in water, as the solubility of methanesulfonates drastically reduces with increasing MSA concentrations, and most salts exhibit very low solubility in MSA solutions that are lean in water. In solutions where MSA concentrations exceed 70 wt%, the maximum concentration of the dissolved metals can fall to less than 1 g L^−1^. The disparity in solubilities between sulfate and methanesulfonate salts is particularly noticeable for lead(ii), calcium, strontium, and barium, while the differences are less pronounced for other metals when considering the aqueous saturation solubility expressed in terms of metal concentration. For instance, 2.16 M of Co^2+^ can be dissolved in the form of CoSO_4_, compared to 2.53 M in the form of Co(CH_3_SO_3_)_2_ (at 22 °C).^5^ Surprisingly, the saturation concentration of Ni^2+^ is even lower when Ni(CH_3_SO_3_)_2_ is dissolved (2.13 M) than when NiSO_4_ is dissolved (2.44 M). All these values must be critically evaluated, recognizing that the formation of double salts can have a significant (negative) impact on the solubility of metal salts. For instance, many transition and rare-earth metals form poorly soluble double sulfate salts with alkali sulfates, while to our best knowledge, no such double salts have been identified for the methanesulfonate salts yet.

A less obvious drawback of MSA pertains to the weakly-coordinating nature of methanesulfonate anions, which results in the absence of neutral ion-pair or anionic methanesulfonate complexes in aqueous solutions. This implies that when extracting metal ions from aqueous solutions *via* solvent extractions, only acidic extractants operating through a cation exchange mechanism can be employed, thereby excluding the use of neutral (solvating) or basic extractants. It is recognized that the use of acidic extractants leads to the consumption of a larger quantity of chemicals, as bases are needed for pH regulation and acids for stripping metals from the loaded organic phase.^206^ The utilization of acids and bases results in the production of substantial volumes of salt waste, often sodium sulfate in solvent extraction processes for battery metals (based on sulfuric acid) and sodium chloride for rare earths (based on hydrochloric acid). In contrast, neutral and basic extractants do not necessitate pH regulation by bases (the controlling factor is the concentration of the anion in the aqueous phase), and stripping can frequently be accomplished simply with water.

Although MSA exhibits lower corrosivity and hazardousness compared to other acids used in industry, it nonetheless presents multiple safety hazards and necessitates cautious handling.^207^ MSA is harmful if ingested or if it comes into contact with the skin, leading to potential severe skin burns and eye damage. Consequently, the utilization of protective gloves, safety attire, and eye protection is obligatory when interacting with MSA solutions, particularly when dealing with concentrated MSA solutions or anhydrous MSA. Prior to initiating work with MSA, it is imperative to consult the Material Safety Data Sheet (MSDS).^208^

## Conclusions and outlook

This Tutorial Review provides an impartial overview of the properties of methanesulfonic acid (MSA) and its applications in clean technologies. The reader is introduced to the advantageous properties of MSA, which include its strong acidity, non-oxidizing nature, high stability against hydrolysis, high redox stability, high chemical stability, low vapor pressure and volatility, colorless and odorless characteristics, biodegradability, and low toxicity. All methanesulfonate salts exhibit high solubility in water, making them highly suitable as electrolytes for electroplating baths and redox flow batteries. MSA can be synthesized with 100% atom economy from methane and sulfur trioxide. When the methane used for MSA synthesis is sourced from biogas, the eco-score of the production process is favorable. If new industrial processes such as the direct sulfonation of methane prove successful, the cost of methanesulfonic acid is expected to decrease further, although it will never be as inexpensive as sulfuric acid.

This means that methanesulfonic acid (MSA) can find applications only in certain areas. It does not make sense to replace sulfuric acid with MSA in those applications where sulfuric acid is currently the undisputed acid of choice, due to price and performance considerations. However, when the coking, dehydrating, or sulfonating properties of concentrated sulfuric acid pose problems in organic synthesis, concentrated MSA can be considered as an alternative. Similarly, when aqueous solutions with high metal concentrations are needed and the corresponding sulfate salts are poorly soluble, highly soluble methanesulfonate salts can be considered. For example, calcium methanesulfonate is highly soluble, whereas calcium sulfate is not. For this reason, MSA electrolytes are used for electroplating, electrowinning, or electrorefining of lead, tin, and lead-solder. MSA also find application as electrolytes for redox flow batteries where high concentrations of metal salts are desired for achieving high energy densities.

While Gernon *et al.* initially presented MSA as a safer and more environmentally friendly alternative for fluoroboric and fluorosilicic acid in electroplating applications,^5^ it is clear from our review that MSA can be used for many more clean technologies.

However, there are still many research questions and ample room for further technological development. For instance, only a few thermodynamic data are available on aqueous methanesulfonate salt electrolytes, and most of these data are for single-metal salts, not for binary or more complex systems. No thermodynamic data exist on methanesulfonate salts in mixed aqueous–organic or organic solvents. The solvent extraction of metal ions from MSA solutions has not been investigated yet. Regarding the applications in hydrometallurgy, it is expected that the future of MSA lies more in the refining and electrowinning of metals, rather than in the leaching of metal ores, and it is very likely that MSA will be used more for recycling metals from spent lithium-ion batteries and other end-of-life products that can be recovered *via* urban mining.

The application of MSA in the electrorefining of copper appears very promising because it allows the attainment of high-purity copper cathodes without the use of organic additives. There is also significant potential for further development of MSA electrolytes for redox flow batteries. Undoubtedly, there will be new applications of MSA that we have not yet envisioned. We eagerly anticipate future exciting research on MSA and its salts in clean technologies.

## Data availability

Since this is a Tutorial Review, no original experimental data have been collected. The references cited in this review can be made available by the corresponding author.

## Conflicts of interest

There are no conflicts to declare.
